# Morphological and Molecular Evidence Reveal Eight New Species of *Gymnopus* from Northeast China

**DOI:** 10.3390/jof8040349

**Published:** 2022-03-28

**Authors:** Jiajun Hu, Guiping Zhao, Yonglan Tuo, Gu Rao, Zhenhao Zhang, Zhengxiang Qi, Lei Yue, Yajie Liu, Tong Zhang, Yu Li, Bo Zhang

**Affiliations:** 1 School of Life Science, Northeast Normal University, Changchun 130024, China; hujjfungi@163.com; 2 Engineering Research Centre of Edible and Medicinal Fungi, Jilin Agricultural University, Ministry of Education, Changchun 130118, China; guipingz6@163.com (G.Z.); tuoyonglan66@163.com (Y.T.); raogufungi@126.com (G.R.); zzhzz34@163.com (Z.Z.); qzx7007@163.com (Z.Q.); yuelei02@126.com (L.Y.); lyj117@163.com (Y.L.); 3 Department of Pesticide and Pesticide Machinery, Jilin Province Agricultural Technology Extension Station, Changchun 130000, China; zt_fungi@163.com

**Keywords:** *Gymnopus* sect. *Levipedes*, *Gymnopus erythropus* complex, new species, Northeast China, phylogenetic analysis

## Abstract

*Gymnopus* is a widely distributed genus consisting of about 300 species thus far, including *Gymnopus fusipes* as a generic type. A total of nine species from China belong to the sect. *Levipedes*, including eight new species—*Gymnopus* *longisterigmaticus*, *Gymnopus* *longus*, *Gymnopus* *macrosporus*, *Gymnopus* *striatus*, *Gymnopus* *changbaiensis*, *Gymnopus* *tomentosus*, *Gymnopus* *tiliicola*, and *Gymnopus* *globulosus*—which were delimited and proposed based on morphological and molecular evidence; and one new record from Jilin Province, China—*Gymnopus erythropus*. Detailed descriptions and illustrations are presented, as well as comparisons to similar species. Overall, our results broaden the morphological characterization of the genus. The pileipellis of sect. *Levipedes* typically takes on the “*Dryophila* structure”, while, in our findings, pileipellis terminal hyphae inflated to spherical to prolate were observed, in addition to extremely long basidia sterigma. The phylogenies inferred from the ITS and nLSU dataset supported the *Gymnopus*, which was defined by Oliveira et al. as a monophyletic genus, and the novel species as separate lineages within. A key to all species described in this study is also provided.

## 1. Introduction

The genus *Gymnopus* (Pers.) Roussel belongs to the family Omphalotaceae, according to Antonín and Noordeloos [1]. There is a long taxonomic research history on this genus, beginning with its proposal by Persoon in 1801 as a tribe of *Agaricus* L. [2]. Later, Fries [3] established *Agaricus* trib. *Collybia* Fr., transferring the species of *Agaricus* trib. *Gymnopus* Pers. into it accordingly. This perspective was widely accepted by other mycologists, until Staude [4] established the genus *Collybia* (Fr.) Staude. Singer [5,6,7] divided the genus *Collybia* into nine sections—sect. *Striipedes* (Fr.) Quél., sect. *Dictyoplocae* (Mont.) Sing., sect. *Iocephalae* Sing. ex Halling, sect. *Levipedes* (Fr.) Quél., sect. *Vestipedes* (Fr.) Quél., sect. *Subfulmosae* Sing., sect. *Cystidiatae* Sing., sect. *Ixotrma* Sing., and sect. *Collybia* Sing.—in his book, *The Agaricales in Modern Taxonomy*. It is the embryonic form of the modern taxonomy of the genus *Collybia* (*Gymnopus*). Based on their research [8,9,10], Halling, Antonín and Noordeloos pointed out that the genus *Collybia* had a problematic and controversial taxonomy and, thus, lacked a clear definition; then Antonín et al. [11] shifted the section and species into *Gymnopus* and *Rhodocollybia* Singer, leaving three species in the genus *Collybia.* The members of *Gymnopus*, in the conception of Antonín and Noordeloos [1,12], are mainly characterized by basidiomata, usually collybioid, marasmioid, and gymnopoid, stipes only rarely arising from the sclerotia, which is a white spore print with smooth basidiospores that are commonly ellipsoid to oblong, typically the presence of clamp connections, a cutis-type pileipellis, ixocutis or similar to a trichoderm, terminal elements mostly coralloid to diverticulate, and usually encrusted pigments. 

With the advent and development of molecular technologies, the phylogenetic analysis of marasmioid and collybioid fungi based on sequences of nuclear ribosomal DNA is just beginning to help clarify generic and infrageneric circumscriptions. Moncalvo et al. [13] pointed out that the genus *Gymnopus* was multiphyletic. Mata et al. [14], who found similar conclusions, stated that *Gymnopus* is more closely related to *Marasmiellus* Murrill. The type specimen of *Marasmiellus*, *Marasmiellus juniperinus* Murrill, was confirmed within *Gymnopus* sect. *Levipedes* (Fr.) Antonín, Halling and Noordel. [12]. More recently, Oliveira et al. [15] redefined the genus *Gymnopus* more strictly standard based on their combined ITS + nLSU phylogenetic analysis. In its conception, the key features of the genus *Gymnopus* are collybioid (rarely tricholomatoid or marasmioid) basidiomata, free, emarginate, or adnate lamellae that are usually crowded, an insititious stipe or not, usually with a strigose base; a white spore print, basidiospores ellipsoid to short-oblong, inamyloid; cheilocystidia usually present, or a variety of cheilocystidia, a cutis or ixocutis pileipellis with radially arranged cylindrical hyphae or interwoven more akin to a trichoderm or ixotrichoderm, made up of irregular coralloid terminal elements (“*Dryophila* structures”)—often incrusted, diverticulate hyphal elements, mixed with broom cells and coralloid hyphae; and clamp connections present in all tissues. As a result, *Gymnopus* sect. *Vestipedes* (Fr.) Antonín, Halling and Noordel. is segregated and placed within *Marasmiellus* s. str, and *Gymnopus* sect. *Perforanita* (Singer) R.H. Petersen is considered a new independent genus *Paragymnopus* J.S. Oliveira. In addition, some *Gymnopus* species were transferred to two new genera [16].

Most species of *Gymnopus* sect. *Levipedes* (Quél.) Halling have smooth, polished, or pubescent stipe; pileipellis mostly as an entangled trichoderm (never radially oriented), composed of inflated, often lobed elements or coralloid (“*Dryophila* structures”); non-dextrinoid trama and elements, with some species turning green in alkali [1,17]. *Gymnopus erythropus* (Pers.) Antonín, Halling and Noordel. is one of the most confusing species in this section. This species was named by Persoon as *Agaricus erythropus* Pers. [2], and then transferred to *Gymnopus* [11]. However, Persoon had a broad conception of this species. Singer [18] selected a neotype from the herbarium of Persoon, labeled *Agaricus erythropus*, which was confirmed to be a *Mycena* (Pers.) Roussel species later on. There remained more specimens labeled *A garicus erythropus* in the herbarium of Persoon, until Jansen [19] studied the material and found that one specimen fit well with the current concept of *Collybia erythropus* (Pers.) P. Kumm. (≡ *Gymnopus erythropus*); considering this, Singer’s choice was rejected. Prior to the current study, only two red stipe species had been reported in this section *Gymnopus erythropus*, and *Gymnopus fagiphilus* (Velen.) Antonín, Halling, and Noordel. These two species are morphologically very similar, with the clear distinguishing factor being the tomentose stipe. Specifically, *Gymnopus erythropus* have a smooth stipe, while those with a dense tomentose at the base of the stipe are *Gymnopus fagiphilus*.

Approximately 300 species have been validly published in the genus *Gymnopus* [20], with most species having been reported from Europe and America. However, research on *Gymnopus* in China is lacking. Teng [21] was the first to report a *Gymnopus* species in China; based on the genus *Collybia*, four taxa were reported. Later, Tai [22] reported eight taxa. Deng [23], a preliminary study on the resources of the genus *Gymnopus* in Southern China, reported 19 taxa. Recently, three new species and 11 new records were recorded from China [23,24,25,26,27,28,29]. Until now, 24 species of *Gymnopus* s. str. have been recognized from China.

This paper aims to describe and illustrate nine species of *Gymnopus* sect. *Levipedes*—eight species new to science, and one new record from Jilin Province, China—based on morphology and molecular studies. 

## 2. Materials and Methods

### 2.1. Sampling and Morphological Studies

The studied specimens were photographed in situ. The size of the basidiomata was measured when fresh. After examination and description of the fresh macroscopic characteristics, the specimens were dried in an electric drier at 40–45 °C.

Descriptions of the macroscopic characteristics were based on field notes and photographs, with the colors corresponding to the *Flora of British fungi: Color identification chart* [30]. The dried specimens were rehydrated in 94% ethanol for microscopic examination, and then mounted in 3% potassium hydroxide (KOH), 1% Congo red (0.1 g Congo red dissolved in 10 mL distilled water), and Melzer’s reagent (1.5 g potassium iodide, 0.5 g crystalline iodine and 22 g chloral hydrate dissolved in 20 mL distilled water) [31]; they were then examined with a Zeiss Axio lab. A1 microscope at magnifications up to 1000×. All measurements were taken from the sections mounted in the 1% Congo red. For each specimen, a minimum of 40 basidiospores, 20 basidia, 20 cheilocystidia, and 20 widths of pileipellis were measured from two different basidiocarps. When reporting the variation in the size of the basidiospores, basidia, cheilocystidia, and width of the pileipellis, 5% of the measurements were excluded from each end of the range, and are given in parentheses. The basidiospores measurements are given as length × width (L × W). Q denotes the variation in the ratio of L to W among the studied specimens, and Qm denotes the average Q value of all the basidiospores ± standard deviation. “I” refers to the number of lamellulae between every two complete lamellae, and “L” refers to the number of complete lamellae. The specimens examined are deposited in the Herbarium of Mycology of Jilin Agricultural University (HMJAU).

### 2.2. DNA Extraction, PCR Amplification, and Sequencing

The total DNA was extracted from dried specimens by using the NuClean Plant Genomic DNA Kit (Kangwei Century Biotechnology Company Limited, Beijing, China), according to the manufacturer’s instructions. Sequences of the internal transcribed spacer (ITS) region, and nuclear large ribosomal subunits (nLSU) were used for phylogenetic analysis. The ITS sequence was amplified by using the primer pair ITS1-F (CTT GGT CAT TTA GAG GAA GTA A) and ITS4-B (CAG GAG ACT TGT ACA CGG TCC AG) [32], and the nLSU sequence was amplified by using the primer pair LROR (GTA CCC GCT GAA CTT AAG C) and LR7 (TAC TAC CAC CAA GAT CT) [33,34]. PCR reactions (25 μL) contained 8 μL 2 × EasyTaq^®^ PCR SuperMix (TransGen Biotech Co., Ltd., Beijing, China), 1 μL 10 μM primer L, 1 μL 10 μM primer R, 3 μL DNA solution, and 12 μL dd H_2_O. The reaction programs were as follows: for the ITS, initial denaturation at 94 °C for 4 min, followed by 30 cycles at 94 °C for 1 min, 54 °C for 1 min and 72 °C for 1 min, and a final extension of 72 °C for 10 min [35]; for the nLSU, initial denaturation at 95 °C for 3 min, followed by 30 cycles at 94 °C for 30 s, 47 °C for 45 s, and 72 °C for 90 s, and a final extension of 72 °C for 10 min [36]. The PCR products were visualized via UV light after electrophoresis on 1% agarose gels stained with ethidium bromide and purified by using the Genview High-Efficiency Agarose Gels DNA Purification Kit (Gen-View Scientific Inc., Galveston, TX, USA). The purified PCR products were then sent to Sangon Biotech Limited Company (Shanghai, China) for sequencing, using the Sanger method. The new sequences were deposited in GenBank (http://www.ncbi.nlm.nih.gov/genbank (accessed on 17 November 2021); see Table 1).

### 2.3. Data Analysis

Based on the results of BLAST and morphological similarities, the sequences obtained and related to these samples were collected and are listed in Table 1. We used a dataset of ITS and nLSU resign comprising sequences from this study, with 49 representative sequences showing the highest similarity to *Gymnopus* spp. This dataset included all *Gymnopus* s. str. section (sect. *Androcacei* (Kühner) Antonín and Noordel., sect. *Levipedes* (Quél.) Halling, sect. *Impudicae* (Antonín and Noordel.) Antonín and Noordel., and sect. *Gymnopus* (Pers.) Roussel) to further explore the relationships of the newly sequenced Chinese specimens within the genus *Gymnopus.* Moreover, the species within this genus and those in allied genera, including *Lentinula* Earle, *Rhodocollybia* Singer, *Mycetinis* Earle, *Marasmiellus* Murrill, *Collybiopsis* (J. Schröt.) Earle, and *Paragymnopus* J.S. Oliveira were included. The sequences of *Marasmius* sect. *Globulares* Kühner, *Marasmius stenophyllus* Mont., *Marasmius aurantioferrugineus* Hongo, *Marasmius brunneospermus* Har. Takah., *Marasmius maximus* Hongo, and *Marasmius nivicola* Har. Takah., were selected as the outgroup taxa [15]. 

For the dataset, each gene region was aligned by using ClustalX [37], MACSE V2.03 [38], or MAFFT 7.490 [39], and then manually adjusted in BioEdit 7.0.5.3 [40]. The datasets first were aligned, and then the ITS and nLSU sequences were combined with Phylosuit V1.2.2 [41]. The best-fit evolutionary model was estimated by using Modelfinder [42]. Following the models, Bayesian inference (BI) algorithms were used to perform the phylogenetic analysis. Specifically, BI was calculated with MrBayes 3.2.6 with a general time-reversible DNA substitution model and a gamma distribution rate variation across the sites [43]. Four Markov chains were run for two runs from random starting trees for two million generations until the split deviation frequency value was <0.01; the trees were sampled every 100 generations. The first 25% of the sampled trees were discarded as burn-in, while all remaining trees were used to construct a 50% majority consensus tree and for calculating the Bayesian posterior probabilities (BPPS). RaxmlGUI 2.0.5 [44] was used for maximum likelihood (ML) analysis, along with 1000 bootstraps (BS) replicates, using the GTRGAMMA algorithm to perform a tree inference and search for the optimal topology [45]. Then the FigTree v1.3.1 was used to visualize the resulting trees.

**Table 1:** Voucher/specimen numbers, country, and GenBank accession numbers of the specimens included in this study. Sequences produced in this study are in bold.

Scientific Name	Country	Voucher/Specimen Numbers	GenBank Accession Numbers		References
			ITS	LSU	
*Collybiopsis dichroa*	USA	TENN56726	AY256702		[46]
*Co. filamentipes*	USA	TENN-F-065861	MN897832	MN897832	[47]
*Co. furtiva*	USA	SFSU: DED4425	DQ450031	AF042650	[47]
*Co. hasanskyensis*	Russia	TENN-F-060730	MN897829		[47]
*Co. juniperina*	USA	TENN59540	AY256708		[14]
*Co. melanopus*	Indonesia	SFSU: A.W. Wilson 54	NR_137539	NG_060624	[48]
*Co. melanopus*	China	LF1758	KU529307		[23]
*Co. mesoamericana*	Costa Rica	TENN 058613	NR_119583	KY019632	[49,50]
*Co. minor*	USA	TENN-F-059993	MN413334	MW396880	[47]
*Co. parvula*	USA	TENN-F-059993	MN413334		Unpublished
*Co. stenophylla*	USA	TENN59449	DQ450033		[46]
*Gymnopus alkalivirens*	USA	TENN51249	DQ450000		[46]
*G. alliifoetidissimus*	China	GDGM76695	MT023344	MT017526	[25]
*G. alpinus*	Latvia	CB16251	JX536168		[51]
*G. androsaceus*	Russia	TENN-F-59594	KY026663	KY026663	[50]
*G. androsaceus*	France	CBS239.53	MH857174	MH868713	[52]
*G. aquosus*	Czech Republic	BRNM665362	JX536172		[51]
*G. aurantiipes*		AWW118	AY263432	AY639410	[48]
*G. bicolor*		AWW116	AY263423	AY639411	[48]
*G. biformis*	USA	TENN58541	DQ450054		[48]
*G. brunneigracilis*		AWW01	AY263434	AY639412	[48]
***G. changbaiensis***	**China**	**HMJAU60300**	**OM030272**	**OM033387**	**this study**
***G. changbaiensis***	**China**	**HMJAU60301**	**OM030273**	**OM033388**	**this study**
***G. changbaiensis***	**China**	**HMJAU60302**	**OM030274**	**OM033389**	**this study**
*G. collybioides*	Costa Rica	TENN58020	AF505772		[46]
*G. confluens*	Sweden	TENN50524	DQ450044		[46]
*G. confluens*	USA	TENN55695	DQ450050		[46]
*G. cylindricus*	Costa Rica	TENN-058097	NR_119464		[49]
*G. densilamellatus*	Republic of Korea	BRNM714984	KP336686	KP336695	[36]
*G. dryophilus*	Czech Republic	BRNM695586	JX536143		[51]
*G. dryophilus*	Germany	BRNM737691	JX536139		[51]
*G. dryophilus*	China	HMAS290095	MK966542		Unpublished
*G. dryophilus*	Japan	Duke31	DQ480099		[46]
*G. dryophioides*	Republic of Korea	BRNM781447	MH589967	MH589985	[53]
*G. dysodes*	USA	TENN59141	AF505778		[46]
*G. erythropus*	Czech Republic	BRNM714784	JX536136		[51]
*G. erythropus*	USA	JFA12910	DQ449998		[46]
*G. erythropus*	Austria	TENN59329	AF505786		[46]
***G. erythropus***	**China**	**HMJAU60313**	**OM030281**	**OM033395**	**this study**
***G. erythropus***	**China**	**HMJAU60315**	**OM030280**	**OM033396**	**this study**
*G. fagiphilus*	Czech Republic	BRNM707079	JX536129		[51]
*G. fusipes*	Austria	TENN59300	AF505777		[46]
*G. fusipes*	France	TENN59217	AY256710	AY256710	[14]
***G. globulosus***	**China**	**HMJAU60307**	**OM030269**	**OM033406**	**this study**
***G. globulosus***	**China**	**HMJAU60308**	**OM030270**	**OM033407**	**this study**
***G. globulosus***	**China**	**HMJAU60308**	**OM030271**	**OM033408**	**this study**
*G. hybridus*	Italy	BRNM695773	JX536177		[51]
*G. inexpectatus*	Italy		EU622905	EU622906	[54]
*G. inusitatus*	Spain	SCM B-4058	JN247553	JN247557	[51]
*G. junquilleus*	USA	TENN55224	NR_119582		[49]
*G. lanipes*	Spain	BRNM670686	JX536137		[51]
***G. longisterigmaticus***	**China**	**HMJAU60288**	**OM030282**	**OM033403**	**this study**
***G. longisterigmaticus***	**China**	**HMJAU60289**	**OM030283**	**OM033404**	**this study**
***G. longisterigmaticus***	**China**	**HMJAU60290**	**OM030284**	**OM033405**	**this study**
***G. longus***	**China**	**HMJAU60291**	**OM030285**	**OM033400**	**this study**
***G. longus***	**China**	**HMJAU60292**	**OM030286**	**OM033401**	**this study**
***G. longus***	**China**	**HMJAU60293**	**OM030287**	**OM033402**	**this study**
***G. macrosporus***	**China**	**HMJAU60294**	**OM030266**	**OM033397**	**this study**
***G. macrosporus***	**China**	**HMJAU60295**	**OM030267**	**OM033398**	**this study**
***G. macrosporus***	**China**	**HMJAU60296**	**OM030268**	**OM033399**	**this study**
*G. ocior*	Czech Republic	BRNM699795	JX536166		[51]
*G. pallipes*	China	GDGM81513	MW582856		[24]
*G. ramulicola*	China	GDGM44256	KU321529		[27]
*G. similis*	Republic of Korea	BRNM766739	KP336692	KP336699	[36]
*G. similis*	China	GDGM78308	MT023352	MT017530	[25]
***G. striatus***	**China**	**HMJAU60297**	**OM030263**	**OM033384**	**this study**
***G. striatus***	**China**	**HMJAU60298**	**OM030264**	**OM033385**	**this study**
***G. striatus***	**China**	**HMJAU60299**	**OM030265**	**OM033386**	**this study**
***G. tiliicola***	**China**	**HMJAU60305**	**OM030275**	**OM033393**	**this study**
***G. tiliicola***	**China**	**HMJAU60306**	**OM030276**	**OM033394**	**this study**
***G. tiliicola***	**China**	**HMJAU60307**	**OM030277**	**OM033392**	**this study**
***G. tomentosus***	**China**	**HMJAU60303**	**OM030278**	**OM033390**	**this study**
***G. tomentosus***	**China**	**HMJAU60304**	**OM030279**	**OM033391**	**this study**
*Letinula aciculospora*	Costa Rica	TENN37996	AY016443		[55]
*L. boryana*	Brazil	TENN58368	AY016440		[55]
*L. edodes*	China	STCL125	AF031183		[56]
*Marasmius aurantioferrugineus*	Republic of Korea	BRNM714752	FJ904962	MK278334	[57]
*M. brunneospermus*	Republic of Korea	KPM-NC0005011	FJ904969	FJ904951	[57]
*M. maximus*	Republic of Korea	BRNM714570	FJ904976	FJ904958	[57]
*M. nivicola*	Republic of Korea	KPM-NC0006038	FJ904973	FJ904955	[57]
*Marasmiellus ramealis*	Sweden	TENN50324	DQ450030		[46]
*Mycetinis. alliaceus*	Russia	TENN-F-55630	KY696784	KY696752	[58]
*My. curraniae*	New Zealand	PDD95301	KY696778		[58]
*My. opacus*	USA	TENN-F-59451	KY696755		[58]
*My. scorodonius*	Switzerland	TENN-F-59451	KY696725		[58]
*Paragymnopus foliiphilus*	USA	TENN-F-68183	KY026705	KY026705	[50]
*P. perforans*	Sweden	TENN-F-50319	KY026625	KY026625	[50]
*P. pinophilus*	USA	TENN-F-69207	KY026725	KY026725	[50]
*Rhodocollyba butyracea*	Sweden	TENN53580	AY313293		[46]
*R. butyracea*	China	HFJAU0269	MN258680		Unpublished
*R. maculata*	Dominican Republic	TFB11720	KT205402		[59]
*R. maculata*	USA	TENN59459	AY313296		[46]

## 3. Results

### 3.1. Phylogenetic Analyses

**Figure 1:**
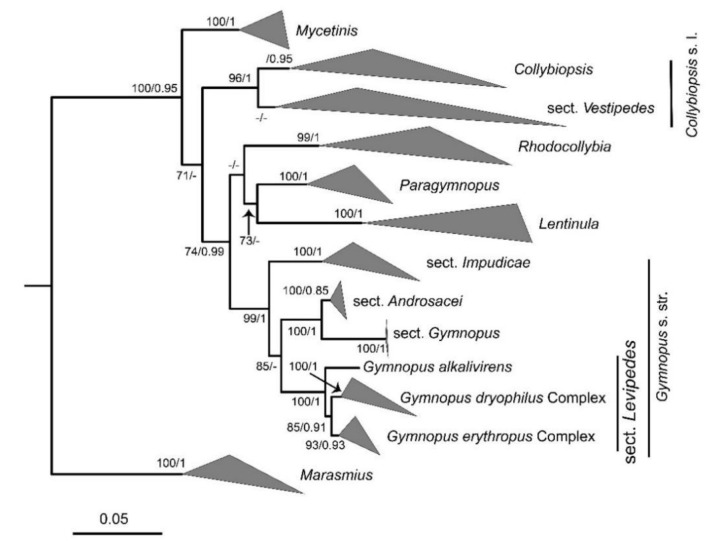
Bayesian 50% majority-rule consensus tree from the ITS and nLSU analyses. Support values at the nodes consist of BPPS ≥ 0.90 and BS ≥ 70; unsupported nodes under BPPS 0.5 are collapsed. The major clade is simplified, representing genus-level groups, as depicted in the figure. The outgroup consists of members of *Marasmius*.

**Figure 2:**
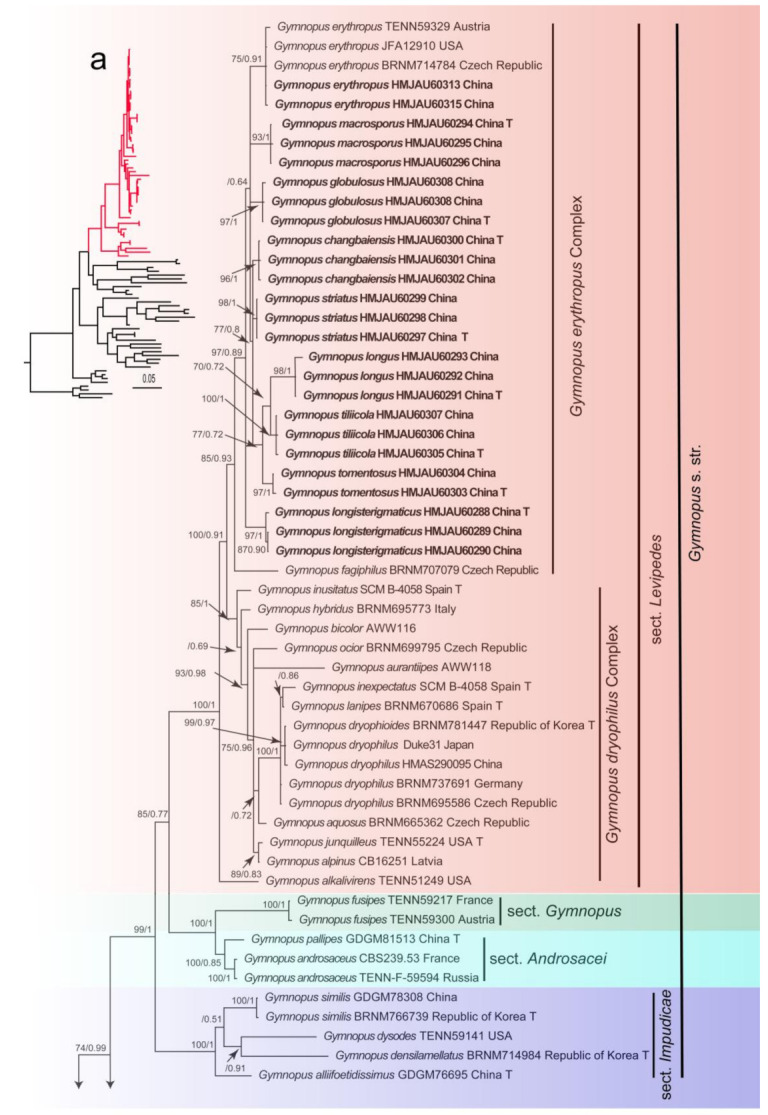

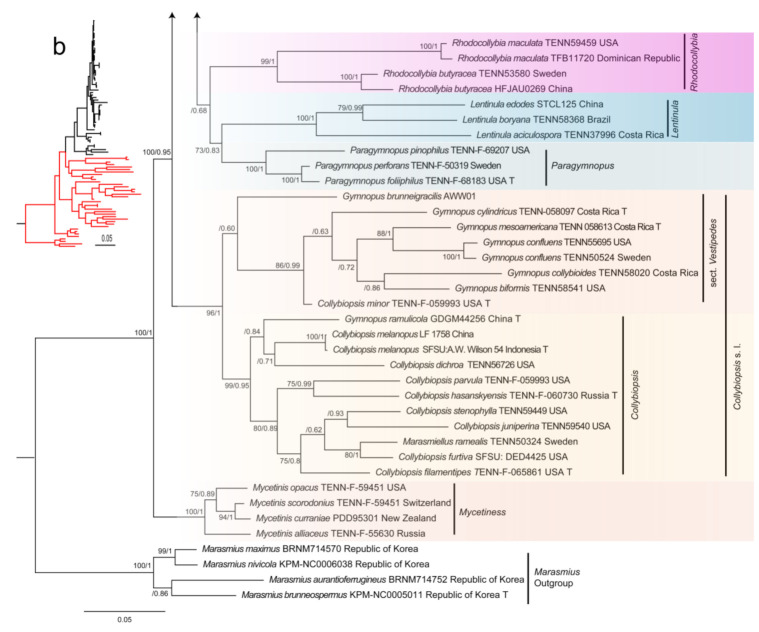
Maximum likelihood phylogenetic tree generated from the ITS and nLSU dataset. Bootstraps values (BS) ≥ 70% from ML analysis and Bayesian posterior probabilities (BPPS) ≥ 0.90 are shown on the branches. Newly sequenced collections are indicated in bold, and the type specimens are denoted by (T).

In the dataset, 143 sequences derived from two gene loci (ITS and nLSU) from 92 samples were used to build phylogenetic trees; 50 of these were newly generated, with 25 ITS sequences and 25 nLSU sequences. The phylogenetic construction performed via ML and BI analysis for the two combined datasets showed a similar topology. The combined ITS and nLSU dataset represented 63 taxa and 2600 characters after being trimmed. The Bayesian analysis was run for two million generations and resulted in an average standard deviation of split frequencies of 0.004989. The same dataset and alignment were analyzed by using the ML method. In the phylogenetic tree, six clades corresponding to *Gymnopus*, *Rhodocollybia*, *Lnetinula*, *Marasmiellus*, *Marasmius*, *Mycetines*, *Collybiopsis*, and *Paragymnopus* were revealed (Figure 1 and Figure 2). Twenty-one sampled specimens formed eight new species and were clustered in a clade comprising the species of *Gymnopus* sect. *Levipedes* (Figure 2). At the same time, two sampled specimens—clustered with *Gymnopus erythropus* with strong support—were confirmed as new records from Jilin Province, China. 

The phylogeny inferred from the dataset of ITS and nLSU region recovered *Gymnopus* s. str. as a monophyletic genus divided into four clades, sect. *Androcacei* clade, sect. *Levipedes* clade, sect. *Impudicae* clade, and sect. *Gymnopus* clade, formed a sister clade to *Rhodocollybia*, *Paragymnopus*, and *Lentinula* (Figure 1). The sect. *Levipedes* clade was mainly divided into three clades, the red stipe species formed an independent clade, and the *Gymnopus dryophilus* complex species formed an independent clade. These two clades mentioned above are near the species *Gymnopus alkalivirens* (Singer) Halling that turns green in KOH, representing *Gymnopus* sect. *Levipedes* subsect. *Alkalivirentes* Antonín and Noorde.

### 3.2. Taxonomy

*Gymnopus longisterigmaticus* J.J. Hu, B. Zhang and Y. Li sp. nov.

**Figure 3:**
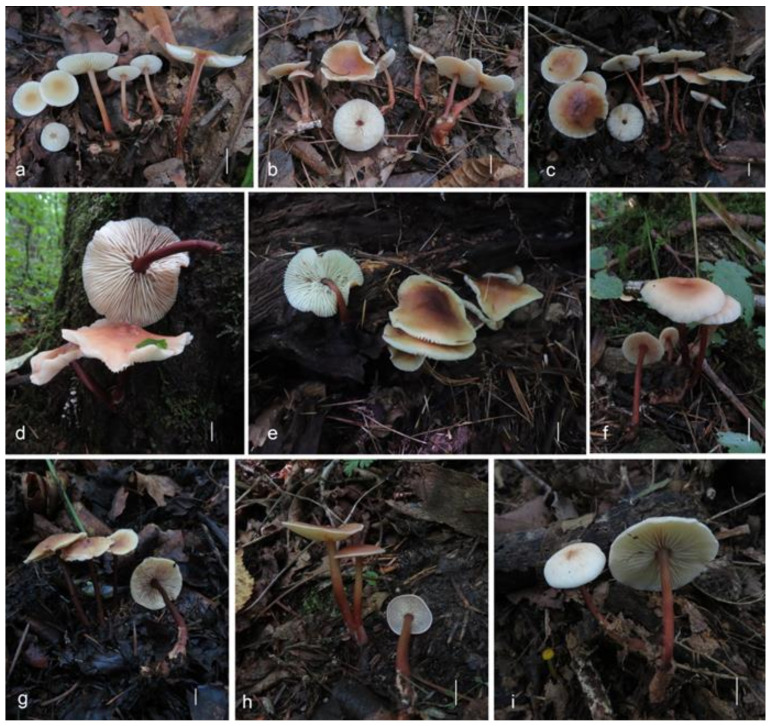
Fresh basidiomata of *Gymnopus* species: (**a**) *Gymnopus longisterigmaticus* (Holotype, HMJAU 60288), (**b**) *Gymnopus longus* (Holotype, HMJAU 60291), (**c**) *Gymnopus macrosporus* (Holotype, HMJAU 60294), (**d**) *Gymnopus tiliicola* (Holotype, HMJAU 60304), (**e**) *Gymnopus globulosus* (Holotype, HMJAU 60308), (**f**) *Gymnopus changbaiensis* (HMJAU 60300), (**g**) *Gymnopus striatus* (Holotype, HMJAU 60297), (**h**) *Gymnopus erythropus* (HMJAU 60315), and (**i**) *Gymnopus tomentosus* (Holotype, HMJAU 60303). Scale bars = 1 cm.

**Figure 4:**
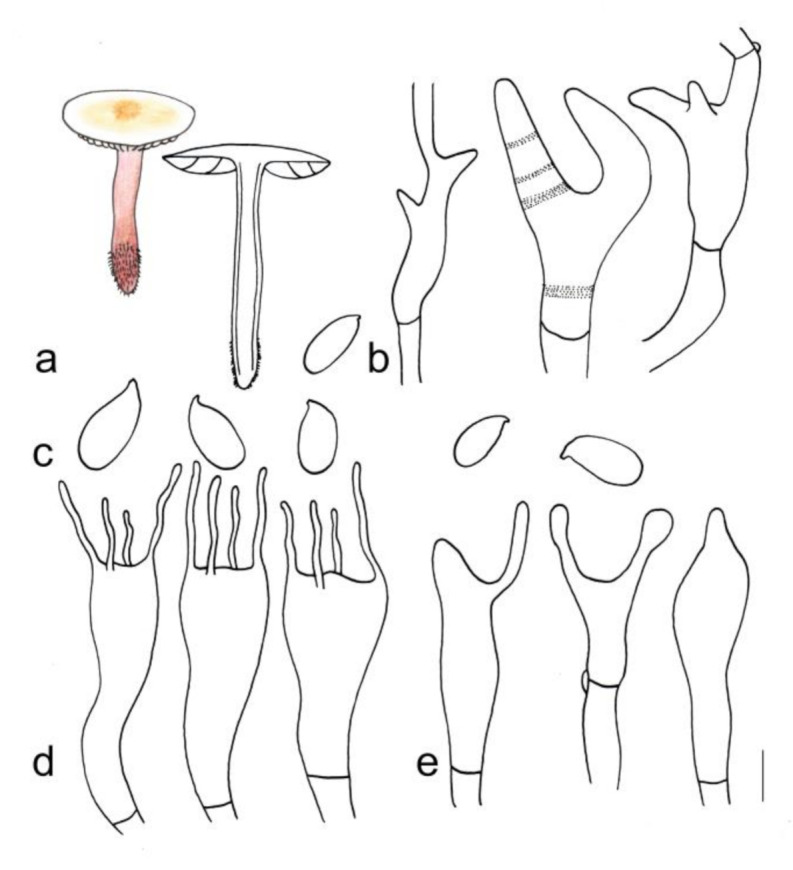
Morphological characteristics of *Gymnopus longisterigmaticus* (HMJAU 60288, holotype): (**a**) basidiomata, (**b**) pileipellis, (**c**) basidiospores, (**d**) basidia, and (**e**) cheilocystidia. Scale bars: 1 cm (**a**), 25 µm (**b**), and 5 µm (**c**–**e**).

Figure 3a and Figure 4

MycoBank: MB 842333

Etymology: The epithet “*longisterigmaticus*” refers to the extremely long sterigmata of the basidia.

Diagnosis: This species is distinguished from closed species by pileus brown at the center, light brown to yellow towards the margin, margin light yellow to yellowish white, stipe reddish brown, covered with white to light reddish brown density hairs at base, basidia four-spored, sterigmata extremely long, pileipellis wider than *Gymnopus longus* and *Gymnopus macrosporus*, branched, pigment yellowish brown incrusting in pileipellis, and larger basidiospores.

Type: China. Jilin Province: Yanbian Korean Autonomous Prefecture, Antu County, Erdaobaihe Town, 42.39° N, 128.11° E, 4 September 2018, Jia-Jun Hu and Bo Zhang, HMJAU 60288, holotype (GenBank accession no.: ITS = OM030282, nLSU = OM033403).

Basidiomata small-to-medium-sized, scattered to gregarious. Pileus convex to applanate, 1.5–3.2 cm diameter, smooth, hygrophanus, brown at the center, light brown to yellow towards the margin, margin light yellow to yellowish white, entire. Context thin, fleshy, light reddish brown, odorless. Stipe center, cylindrical, 3.2–5.0 cm long and 0.2–0.3 cm wide, reddish brown, smooth, covered with white to light reddish brown density hairs at base, fistulose, fibrous. Lamellae subfree to adnate, white to light yellow, I = 1–3, L = 15–18, crowded.

Basidiospores elliptic, (6.2) 6.7–9.0 × (3.0) 3.1–4.3 (5.0) µm, Q = (1.40) 1.67–2.25 (2.26), Qm = 1.93 ± 0.20, smooth, hyaline, inamyloid, thin-walled. Basidia clavate, (18) 19–27 (28) × (5) 6–10 µm, four-spored, thin-walled, smooth, hyaline; sterigmata extremely long, up to 40 µm. Cheilocystidia abundant, clavate, with obtuse on the top, or branched, (16) 18–27 × (4) 5–8 (9) µm, thin-walled, smooth, hyaline. Pileipellis a cutis, made up of irregularly branched hyphae, inflated, 10–27 (35) µm wide, hyaline to light yellow, smooth or pigment yellowish brown incrusting in pileipellis, thin-walled, clamps present. 

Ecology: Grows on the deciduous layer or rotten branches in coniferous and broad-leaved mixed forest.

Distribution: China (Jilin Province)

Other specimen examined: China. Jilin Province: Yanbian Korean Autonomous Prefecture, Antu County, Erdaobaihe Town, 42.39° N, 128.11° E, 13 September 2019, Jia-Jun Hu and Bo Zhang, HMJAU 60289 (GenBank accession no.: ITS = OM030283, nLSU = OM033404); Yanbian Korean Autonomous Prefecture, Antu County, Erdaobaihe Town, 42.39° N, 128.11° E, 13 September 2019, Jia-Jun Hu and Bo Zhang, HMJAU 60290 (GenBank accession no.: ITS = OM030284, nLSU = OM033405).

Note: Morphologically, *Gymnopus longisterigmaticus* is similar to *Gymnopus erythropus* and *Gymnopus fagiphilus* with its reddish brown stipe. However, *Gymnopus longisterigmaticus* differs from *Gymnopus erythropus* with its light reddish brown density hairs on the stipe, extremely long sterigmata of basidiomata (up to 40 µm), different shape of cheilocystidia—cheilocystidia of *Gymnopus erythropus* is clavate to subclavate or somewhat flexuous, coralloid at apex sometimes [1], while clavate of *Gymnopus longisterigmaticus*, and quite larger basidiospores [(6.2) 6.7–9.0 × (3.0) 3.1–4.3 (5.0) µm]. 

*Gymnopus longisterigmaticus* and *Gymnopus fagiphilus* are both covered with hairs on the stipe, but the lamellae of *Gymnopus longisterigmaticus* is white to light yellow, while that of *Gymnopus fagiphilus* is pinkish brown to pinkish yellow; on the other hand, *Gymnopus longisterigmaticus* has extremely long sterigmata of the basidia and lack of chaulocystidia. Moreover, the different shape and size of cheilocystidia can differentiate *Gymnopus longisterigmaticus* from *Gymnopus fagiphilus*. The cheilocystidia of *Gymnopus fagiphilus* is usually irregularly clavate, often with lobed apex or with short-to-long rostrum, sometimes very slender and lageniform and quite larger [15–40 (60) × 4.0–8.0 (10) μm] [1], while cheilocystidia of *Gymnopus longisterigmaticus* is clavate, branched or obtuse. 

*Gymnopus longus* J.J. Hu, B. Zhang and Y. Li sp. nov.

**Figure 5:**
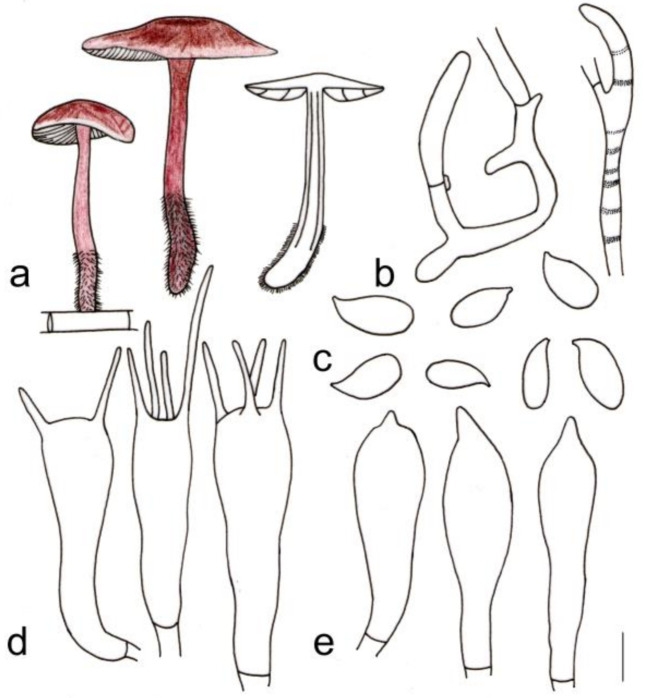
Morphological characteristics of *Gymnopus longus* (HMJAU 60291, holotype): (**a**) basidiomata, (**b**) pileipellis, (**c**) basidiospores, (**d**) basidia, and (**e**) cheilocystidia. Scale bars: 1 cm (**a**), 25 µm (**b**), and 5 µm (**c**–**e**).

Figure 3b and Figure 5

MycoBank: MB 842334

Etymology: The epithet “*longus*” refers to the extremely long sterigmata of the basidia.

Diagnosis: *Gymnopus longus* can be easily differentiated from closely-related species *Gymnopus fagiphilus* by its pileus reddish brown, stipe reddish brown, with brown farinose on the upper part and white to light reddish brown tomentose at the base, basidia 2- or 4-spored, sterigmata extremely long, and smaller basidiospores.

Type: China. Jilin Province: Yanbian Korean Autonomous Prefecture, Antu County, Erdaobaihe Town, 42.39° N, 128.11° E, 4 September 2018, Jia-Jun Hu, Bo Zhang, and Gui-Ping Zhao, HMJAU 60291, holotype (GenBank accession no.: ITS = OM030285, nLSU = OM033400).

Basidiomata small-to-medium-sized, scattered to gregarious. Pileus 1.7–3.7 cm in diameter, convex to applanate or revolute, smooth, hygrophanus, reddish brown at the center, towards margin light reddish brown to brown; margin white to light yellow or light brown, entire. Context thin, fleshy, light reddish brown, odorless. Stipe center, cylindrical to clavate, 3.7–4.3 cm long and 0.3–0.6 cm wide, reddish brown, with brown farinose on the upper part, and white to light reddish brown tomentose at the base, hollow, filiform. Lamellae adnate, white to light yellow, I = 5–7, L = 19–24, crowded. Spores print unknown.

Basidiospores (5.6) 6.0–8.0 × (3.0) 3.1–4.1 (4.9) µm, Q = (1.27) 1.47–2.19 (2.58), Qm = 1.8 ± 0.24, oblong, smooth, hyaline, thin-walled, inamyloid. Basidia (19) 20–28 (29) × 6–9 µm, two- or four-spored, hyaline, thin-walled, clavate; sterigmata extremely long, up to 33 µm long. Cheilocystidia (21) 22–29 (30) × 5–7 µm, mass, clavate, with obtuse on the top, hyaline, thin-walled, smooth. Pileipellis a translation between a cutis and a trichoderm, made up of irregularly interwoven, repent or ascending, inflated hyphae with inflated and irregularly branched terminal elements, hyaline to light brown, (6) 7–13 (15) µm wide, smooth or pigment yellowish brown incrusting in pileipellis.

Ecology: Grows on the deciduous layer or rotten branches in coniferous and broad-leaved mixed forest.

Other specimen examined: China. Jilin Province: Yanbian Korean Autonomous Prefecture, Antu County, Erdaobaihe Town, 42.39° N, 128.11° E, 4 September 2018, Jia-Jun Hu, Bo Zhang, and Gui-Ping Zhao, HMJAU 60292 (GenBank accession no.: ITS = OM030286, nLSU = OM033401); Yanbian Korean Autonomous Prefecture, Antu County, Erdaobaihe Town, 42.39° N, 128.11° E, 31 August 2020, Jia-Jun Hu, Bo Zhang, and Gui-Ping Zhao, HMJAU 60293 (GenBank accession no.: ITS = OM030287, nLSU = OM033402).

Note: *Gymnopus longus* is closed to *Gymnopus erythropus*, *Gymnopus fagiphilus*, and *Gymnopus longisterigmaticus* in morphological, because of the red pileus and stipe. However, *Gymnopus longus* differs from *Gymnopus erythropus* by being covered with brown farinose on the upper part, white to light reddish brown tomentose at the base, slight thin basidiospores, smaller Qm [1], and extremely long sterigmata (up to 33 µm long). 

A deeper color pileus, covered with brown farinose on the stipe, smaller basidiospores, clavate with obtuse cheilocystidia, and pileipellis a translation between a cutis and a trichoderm differs *Gymnopus longisterigmaticus* from *Gymnopus longus*. *Gymnopus longus* differs from *Gymnopus fagiphilus* by a farinose stipe, deep color pileus and stipe, white lamellae, smaller basidiospores, lack of caulocystidia, and uncoralloid pileipellis [1].

*Gymnopus macrosporus* J.J. Hu, B. Zhang and Y. Li sp. nov.

**Figure 6:**
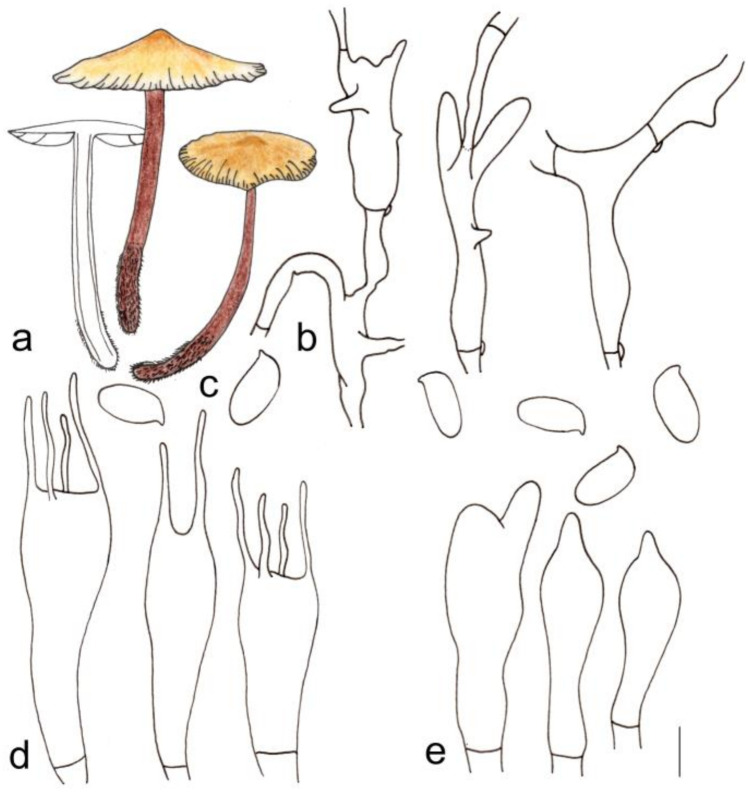
Morphological characteristics of *Gymnopus macrosporus* (HMJAU 60294, holotype): (**a**) basidiomata, (**b**) pileipellis, (**c**) basidiospores, (**d**) basidia, and (**e**) cheilocystidia. Scale bars: 1 cm (**a**), 25 µm (**b**), and 5 µm (**c**–**e**).

Figure 3c and Figure 6

MycoBank: MB 842335

Etymology: the epithet “*macrosporus*” refers to the big basidiospores of this species.

Diagnosis: This species is distinguished from closed species by a convex to applanate pileus that is deep reddish brown at the center and reddish brown to yellowish brown toward the margin, with the margin beige to light yellow, striped; a deep reddish brown to reddish brown stipe with smooth, light yellow to light reddish brown tomentose at the base, coralloid pileipellis, bigger basidiospores, and extremely long basidia sterigmata. 

Type: China. Jilin Province: Yanbian Korean Autonomous Prefecture, Antu County, Erdaobaihe Town, 42.39° N, 128.11° E, 4 September 2018, Jia-Jun Hu and Bo Zhang, HMJAU 60294, holotype (GenBank accession no.: ITS = OM030266, nLSU = OM033397).

Basidiomata small-to-medium-sized, gregarious. Pileus convex to applanate, 1.2–4.6 cm diameter, smooth, hygrophanus, deep reddish brown at the center, reddish brown to yellowish brown towards margin; margin beige to light yellow, striped, entire, wavy. Context thin, fleshy, light reddish brown to light yellowish brown, odorless. Stipe center, cylindrical, 7.8–9.5 cm long and 0.2–0.5 cm wide, deep reddish brown to reddish brown, smooth, fistulose, fibrous, and light yellow to light reddish brown tomentose at the base. Lamellae adnexed to adnate or near free, light yellow, I = 1–3, L = 13–17, crowded.

Basidiospores elliptic, (6.0) 6.8–7.9 (8.3) × (3.0) 3.1–4.2 (4.3) µm, Q = (1.63) 1.67–2.32 (2.37), Qm = 1.88 ± 0.18, smooth, hyaline, inamyloid, thin-walled. Basidia clavate, 20–29 × 6–9 µm, two- or four-spored, thin-walled, smooth, hyaline; sterigmata extremely long, up to 32 µm. Cheilocystidia abundant, clavate, with obtuse on the top, 20–28 (30) × 5 (6)–9 µm, thin-walled, smooth, hyaline. Pileipellis a cutis, made up of irregular branched or weakly coralloid hyphae, inflated, 10–27 (35) µm wide, hyaline to light yellow, smooth, thin-walled, clamps present. 

Ecology: Grows on the deciduous layer or rotten branches in coniferous and broad-leaved mixed forest.

Distribution: China (Jilin Province)

Other specimen examined: China. Jilin Province: Yanbian Korean Autonomous Prefecture, Antu County, Erdaobaihe Town, 42.39° N, 128.11° E, 4 September 2018, Jia-Jun Hu and Bo Zhang, HMJAU 60295 (GenBank accession no.: ITS = OM030267, nLSU = OM033398); Yanbian Korean Autonomous Prefecture, Antu County, Erdaobaihe Town, 42.39° N, 128.11° E, 13 September 2019, Jia-Jun Hu and Bo Zhang, HMJAU 60296 (GenBank accession no.: ITS = OM030268, nLSU = OM033399).

Note: *Gymnopus macrosporus* is morphologically similar to *Gymnopus longisterigmaticus* and *Gymnopus longus* because of its reddish brown, tomentose stipe, and long sterigmata of basidia. *Gymnopus macrosporus* differs from *Gymnopus longus* due to its pileus in a darker color—pileus deep reddish brown at center, reddish brown to yellowish brown towards margin; margin beige to light yellow, striped characteristics, and smooth texture on the upper part of the stipe, coralloid and without pigment incrusting pileipellis. These two *Gymnopus* species have a similar basidiospore size; however, the Qm of *Gymnopus macrosporus* is larger than *Gymnopus longus*. *Gymnopus longisterigmaticus* differs in smooth, pale color, and unstriped pileus; pileipellis a bit wider and pigment yellowish brown incrusting in pileipellis, and it has bigger basidiospores [(6.2) 6.7–9.0 × (3.0) 3.1–4.3 (5.0) μm].

*Gymnopus striatus* J.J Hu, B. Zhang and Y. Li sp. nov.

**Figure 7:**
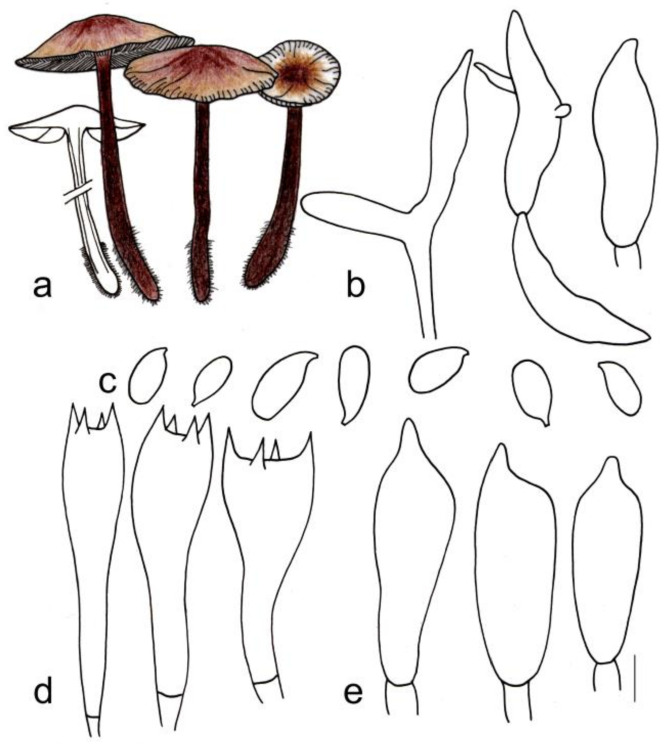
Morphological characteristics of *Gymnopus striatus* (HMJAU 60297, holotype): (**a**) basidiomata, (**b**) pileipellis, (**c**) basidiospores, (**d**) basidia, and (**e**) cheilocystidia. Scale bars: 1 cm (**a**), 25 µm (**b**), and 5 µm (**c**–**e**).

Figure 3g and Figure 7

MycoBank: MB 842336

Etymology: the epithet “*striatus*” refers to the striped stipe of this species.

Diagnosis: This species is distinguished from closed species by a cinnamon pileus, with a lighter color toward the margin and a white to light yellow margin, striped; yellow to light brown lamellae, a deep reddish brown stipe, longitudinal striped stipe up to 1/3 covered with yellow to light brown hairs (from the base upward), short sterigmata of basidia, and smaller basidiospores.

Type: China. Jilin Province: Yanbian Korean Autonomous Prefecture, Antu County, Erdaobaihe Town, 42.39° N, 128.11° E, 9 September 2019, Jia-Jun Hu, Gui-Ping Zhao, and Bo Zhang, HMJAU 60297, holotype (GenBank accession no.: ITS = OM030263, nLSU = OM033384).

Basidiomata small-to-medium-sized, gregarious. Pileus convex to applanate, depressed when old, 2.3–4.1 cm diameter, smooth, hygrophanus, cinnamon at the center, brown to light brown towards margin; margin white to light yellow, striped, entire, wavy. Context thin, fleshy, light yellowish brown, odorless. Stipe center, cylindrical to clavate, 5.5–7.0 cm long and 0.3–0.8 cm wide, deep reddish brown to reddish brown, smooth in the upper part, longitudinal striped, covered with yellow to light brown hairs up to 1/3 (from the base upwards), fistulose, fibrous. Lamellae adnate, yellow to light brown, I = 3–9, L = 17–23, crowded.

Basidiospores elliptic, 6.0–8.0 (9.0) × 3.0–4.0 µm, Q = (1.50) 1.58–2.50 (2.60), Qm = 2.01 ± 0.25, smooth, hyaline, inamyloid, thin-walled. Basidia clavate, 20 (21)–34 (37) × 5–10 µm, two- or four-spored, thin-walled, smooth, hyaline. Cheilocystidia abundant, clavate, with obtuse on the top, (17) 20–30 × 4–8 (10) µm, thin-walled, smooth, hyaline. Pileipellis a cutis, made up of irregular branched or weakly coralloid hyphae, inflated, 10–30 (35) µm wide, hyaline to light yellow, smooth, thin-walled, clamps present. 

Ecology: Grows on the deciduous layer or rotten branches in coniferous and broad-leaved mixed forest.

Distribution: China (Jilin Province)

Other specimen examined: China. Jilin Province: Yanbian Korean Autonomous Prefecture, Antu County, Erdaobaihe Town, 42.39° N, 128.11° E, 18 September 2020, Jia-Jun Hu, Gui-Ping Zhao, and Bo Zhang, HMJAU 60298 (GenBank accession no.: ITS = OM030264, nLSU = OM033385); Yanbian Korean Autonomous Prefecture, Antu County, Erdaobaihe Town, 42.39° N, 128.11° E, 18 September 2020, Jia-Jun Hu, Gui-Ping Zhao, and Bo Zhang, HMJAU 60299 (GenBank accession no.: ITS = OM030265, nLSU = OM033386).

Note: *Gymnopus striatus* is easily confused with *Gymnopus longisterigmaticus*, *Gymnopus longus*, and *Gymnopus macrosporus* due to their highly similar morphology. However, *Gymnopus striatus* differs from those three species by its deeper color lamellae, longitudinal stripes on the stipe and stripes on the margin of pileus, bigger Qm, and short basidia sterigmata. *Gymnopus striatus* can be easily differentiated from *Gymnopus fagiphilus* by its deeper color pileus, uniform colored and longitudinally striped stipe, lack of caulocystidia, uncoralloid cheilocystidia, without pigment incrusting in pileipellis, and smaller basidiospores.

*Gymnopus changbaiensis* J.J. Hu, B. Zhang and Y. Li sp. nov.

**Figure 8:**
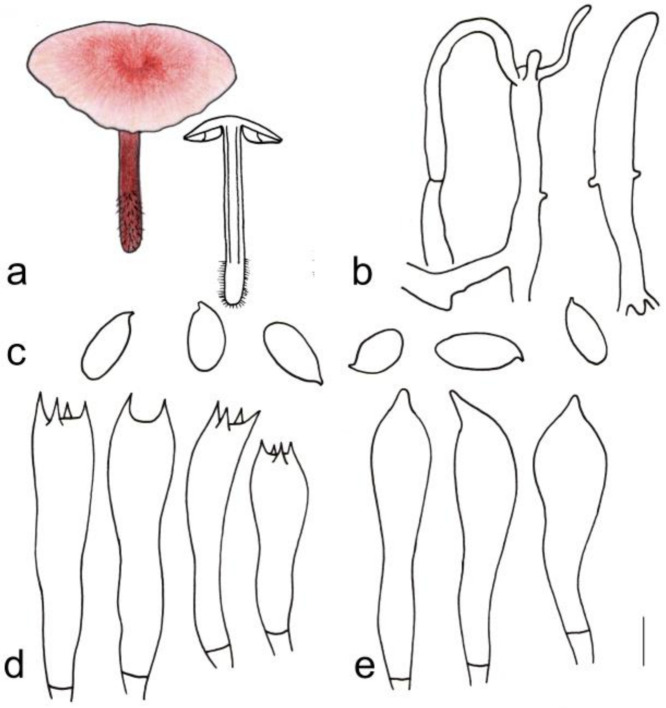
Morphological characteristics of *Gymnopus changbaiensis* (HMJAU 60300, holotype): (**a**) basidiomata, (**b**) pileipellis, (**c**) basidiospores, (**d**) basidia, and (**e**) cheilocystidia. Scale bars: 1 cm (**a**), 25 µm (**b**), and 5 µm (**c**–**e**).

Figure 3f and Figure 8

MycoBank: MB 842337

Etymology: the epithet “*changbaiensis*” refers to Mt. Changbai, the location of the holotype.

Diagnosis: This species is distinguished from closed species by a reddish brown pileus and depressed when mature at the center, light pink to white outwards and margin, striped; fresh to pink lamellae, and a reddish brown stipe up to 1/3 covered with light yellow to brown hairs (from the base upwards), short sterigmata of basidia, lack of caulocystidia, uncoralloid cheilocystidia and smaller basidiospores.

Type: China. Jilin Province: Baishan City, Changbai Korean Autonomous County, Wangtian’e Scenic Spot, 41.56° N, 127.95° E, 17 September 2020, Jia-Jun Hu, Gui-ping Zhao, and Bo Zhang, HMJAU 60300, holotype (GenBank accession no.: ITS = OM030272, nLSU = OM033387).

Basidiomata small-to-medium-sized, gregarious. Pileus hemispherical, deep reddish brown when young, convex or slightly depressed sometimes when mature, 2.1–3.4 cm diameter, smooth, hygrophanus, reddish brown at the center, light pink towards margin; margin white to light pink, striped, entire. Context thin, fleshy, light yellowish brown, odorless. Stipe center, cylindrical, 4.2–5.3 cm long and 0.2–0.3 cm wide, deep reddish brown to reddish brown, smooth in the upper part, covered with light yellow to brown hairs up to 1/3 (from the base upwards), fistulose, fibrous. Lamellae adnate, fresh to pink, I = 1–5, L = 19–24, crowded.

Basidiospores elliptic, (5.8) 6.0–8.1 (9.0) × 3.0–4.1 (4.2) µm, Q = (1.41) 1.53–2.40 (2.50), Qm = 1.98 ± 0.24, smooth, hyaline, inamyloid, thin-walled. Basidia clavate, (19) 20–29 (32) × 5–8 µm, two- or four-spored, thin-walled, smooth, hyaline. Cheilocystidia abundant, clavate, with obtuse on the top, (23) 24–34 (39) × (5) 6–7 (9) µm, thin-walled, smooth, hyaline. Pileipellis a cutis, made up of irregular branched or weakly coralloid hyphae, inflated, 8–23 (25) µm wide, hyaline to light yellow, smooth, thin-walled, clamps present. 

Ecology: Grows on the deciduous layer or rotten branches in coniferous and broad-leaved mixed forest.

Distribution: China (Jilin Province)

Other specimen examined: China. Jilin Province: Baishan City, Changbai Korean Autonomous County, Wangtian’e Scenic Spot, 41.56° N, 127.95° E, 9 September 2019, Jia-Jun Hu, Gui-ping Zhao, and Bo Zhang, HMJAU 60301 (GenBank accession no.: ITS = OM030273, nLSU = OM033388); Baishan City, Changbai Korean Autonomous County, Wangtian’e Scenic Spot, 41.56° N, 127.95° E, 9 September 2019, Jia-Jun Hu, Gui-ping Zhao, and Bo Zhang, HMJAU 60302 (GenBank accession no.: ITS = OM030274, nLSU = OM033389).

Note: *Gymnopus changbaiensis* is significantly related to *Gymnopus fagiphilus* and *Gymnopus striatus* based on its reddish brown and tomentose stipe, and short basidia sterigmata. *Gymnopus changbaiensis* can be distinguished from *Gymnopus fagiphilus* by its lighter and depressed pileus, denser and fresh to pink lamellae, and, in terms of microscopic characteristics, smaller basidiospores, uncoralloid cheilocystidia, and lack of caulocystidia. *Gymnopus changbaiensis* differs from *Gymnopus striatus* by its pale color, striped, and depressed pileus, fresh-to-pink lamellae, non-striped stipe, a bit longer cheilocystidia, coralloid pileipellis. 

*Gymnopus tomentosus* J.J. Hu, B. Zhang and Y. Li sp. nov.

**Figure 9:**
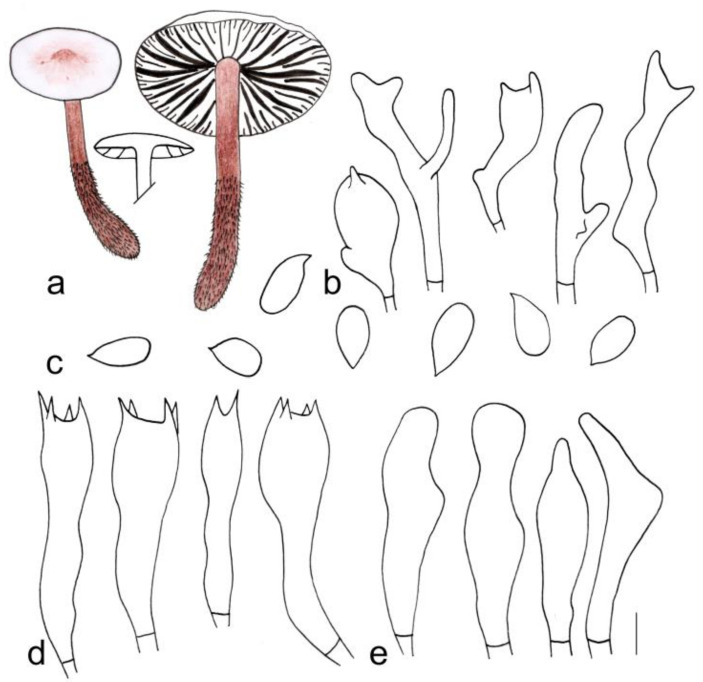
Morphological characteristics of *Gymnopus tomentosus* (HMJAU 60303, holotype): (**a**) basidiomata, (**b**) pileipellis, (**c**) basidiospores, (**d**) Basidia, and (**e**) cheilocystidia. Scale bars: 1 cm (**a**), 25 µm (**b**), and 5 µm (**c**–**e**).

Figure 3i and Figure 9

MycoBank: MB 842338

Etymology: the epithet “*tomentosus*” refers to the tomentose margin of pileus.

Diagnosis: This species is distinguished from closed species by a near white pileus with a tomentose margin, yellowish green lamellae, and a reddish brown stipe up to 1/4 covered with reddish brown hairs (from the base upwards), smaller basidiospores, clavate cheilocystidia, and inflated to bulbous pileipellis. 

Type: China: Jilin Province, Jiaohe City, Lafa Mountain National Forest Park Red Leaf Valley Scenic Spot, 43.71° N, 127.08° E, 7 September 2019, Jia-Jun Hu, Gui-ping Zhao, and Bo Zhang, HMJAU 60303, holotype (GenBank accession no.: ITS = OM030278, nLSU = OM033390).

Basidiomata small-to-medium-sized, scattered. Pileus convex, 1.6–3.0 cm diameter, smooth, tan at the center, light brown towards margin; margin white, tomentose, entire. Context thin, fleshy, white to light yellow, odorless. Stipe center, cylindrical, 3.3–4.3 cm long and 0.2–0.5 cm wide, blackish green at apex, reddish brown below, covered with reddish brown hairs up to 1/4 (from the base upwards), fistulose, fibrous. Lamellae adnexed, yellowish green, I = 3–7, L = 19–25, crowded.

Basidiospores elliptic, (6.0) 6.2–8.2 (9.0) × 3.0–4.1 (4.2) µm, Q = (1.50) 1.59–2.33 (2.40), Qm = 1.92 ± 0.23, smooth, hyaline, inamyloid, thin-walled. Basidia clavate, 20–30 (31) × 5–8 µm, two- or four-spored, thin-walled, smooth, hyaline. Cheilocystidia abundant, clavate, with obtuse on the top sometimes, (20) 22–30 (32) × 5–7 µm, thin-walled, smooth, hyaline. Pileipellis a cutis, made up of irregular branched to weakly coralloid or bulbous hyphae, inflated, 10–18 (21) µm wide, light brown, smooth, thin-walled, clamps present. 

Ecology: Grows on the deciduous layer in broad-leaved forest.

Distribution: China (Jilin Province)

Note: The reddish brown and tomentose stipe makes *Gymnopus tomentosus* similar to *Gymnopus fagiphilus*, *Gymnopus longisterigmaticus*, *Gymnopus longus*, *Gymnopus macrosporus, Gymnopus striatus*, and *Gymnopus changbaiensis*. However, its white-to-pale-yellow pileus with a tomentose margin and inflated bulbous terminal hyphae of the pileipellis differentiates *Gymnopus tomentosus* from *Gymnopus longisterigmaticus*, *Gymnopus longus*, *Gymnopus macrosporus*, *Gymnopus striatus*, and *Gymnopus changbaiensis*. *Gymnopus tomentosus* can be distinguish from *Gymnopus fagiphilus* by its near-white pileus with a tomentose margin, coralloid-to-bulbous pileipellis, smaller basidiospores, and lack of caulocystidia [1].

*Gymnopus tiliicola* J.J. Hu, B. Zhang and Y. Li sp. nov.

**Figure 10:**
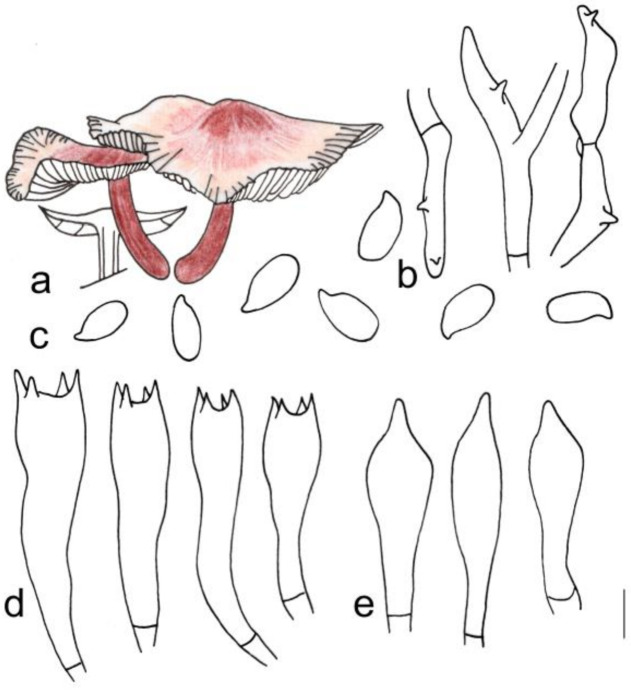
Morphological characteristics of *Gymnopus tiliicola* (HMJAU 60304, holotype): (**a**) basidiomata, (**b**) pileipellis, (**c**) basidiospores, (**d**) basidia, and (**e**) cheilocystidia. Scale bars: 1 cm (**a**), 25 µm (**b**), and 5 µm (**c**–**e**).

Figure 3d and Figure 10

MycoBank: MB 842339

Etymology: the epithet “*tiliicola*” refers to this species grows at the base of *Tilia* sp.

Diagnosis: This species is distinguished from closed species by a deep rose-red pileus that is pale color outward, light pink to fresh lamellae, with a deep reddish brown and smooth stipe that is longitudinally striped, grows at the base of *Tilia* sp., uncoralloid cheilocystidia, two- or four-spored basidia, and a bit bigger basidiospores.

Type: China. Jilin Province: Yanbian Korean Autonomous Prefecture, Antu County, Erdaobaihe Town, 42.39° N, 128.11° E, 13 September 2019, Jia-Jun Hu, Gui-Ping Zhao, and Bo Zhang, HMJAU 60304, holotype (GenBank accession no.: ITS = OM030275, nLSU = OM033392).

Basidiomata medium-to-large-sized, gregarious. Pileus convex, 3.0–6.7 cm diameter, smooth, deep rose-red at the center, yellowish pink towards margin; margin white to light yellow, striped, entire, wavy. Context thin, fleshy, white to pink, odorless. Stipe center, cylindrical, 2.2–4.5 cm long and 0.3–0.7 cm wide, deep reddish brown, smooth, fistulose, fibrous. Lamellae adnexed to adnate, light pink to fresh, I = 1–3, L = 19–24, crowded.

Basidiospores elliptic, (6.0) 6.9–8.0 (8.2) × (3.0) 3.1–4.0 (4.2) µm, Q = (1.70) 1.75–2.26 (2.33), Qm = 1.93 ± 0.17, smooth, hyaline, inamyloid, thin-walled. Basidia clavate, 20–30 × 6–8 µm, two- or four-spored, thin-walled, smooth, hyaline. Cheilocystidia abundant, clavate, with obtuse on the top, (20) 21–27 (28) × 5–7 µm, thin-walled, smooth, hyaline. Pileipellis a cutis, made up of irregular branched to weakly coralloid hyphae, inflated, (5) 6–15 (17) µm wide, light brown, smooth, thin-walled, clamps present. 

Ecology: Grows at the base of *Tilia* sp.

Distribution: China (Jilin Province)

Other specimen examined: China. Jilin Province: Yanbian Korean Autonomous Prefecture, Antu County, Erdaobaihe Town, 42.39° N, 128.11° E, 31 August 2020, Jia-Jun Hu, Gui-Ping Zhao, and Bo Zhang, HMJAU 60305 (GenBank accession no.: ITS = OM030277, nLSU = OM033393); Yanbian Korean Autonomous Prefecture, Antu County, Erdaobaihe Town, 42.39° N, 128.11° E, 27 August 2021, Jia-Jun Hu, Gui-Ping Zhao, and Bo Zhang, HMJAU 60304 (GenBank accession no.: ITS = OM030276, nLSU = OM033394).

Note: Morphologically, the rose-red to dark red pileus and stipe make *Gymnopus tiliicola* closed to *Gymnopus erythropus*. *Gymnopus tiliicola* differs from *Gymnopus erythropus* in a lighter color and striped pileus, light pink to fresh and denser lamellae. Besides, *Gymnopus tiliicola* grows at the base of *Tilia* sp., while *Gymnopus erythropus* grows on the deciduous layer or rotten branches. In regard to microfeatures, *Gymnopus tiliicola* differs from *Gymnopus erythropus* by a weakly coralloid pileipellis, uncoralloid cheilocystidia, bigger basidiospores, and two- or four-spored basidia. 

*Gymnopus globulosus* J.J. Hu, Y.L. Tuo, B. Zhang and Y. Li sp. nov.

**Figure 11:**
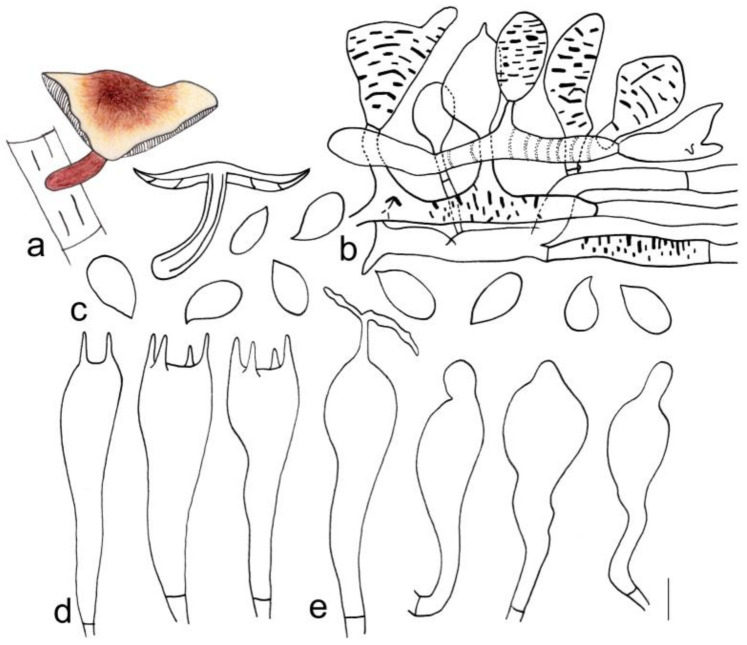
Morphological characteristics of *Gymnopus globulosus* (HMJAU 60307, holotype): (**a**) basidiomata, (**b**) pileipellis, (**c**) basidiospores, (**d**) basidia, and (**e**) cheilocystidia. Scale bars: 1 cm (**a**), 25 µm (**b**), and 5 µm (**c**–**e**).

Figure 3e and Figure 11

MycoBank: MB 842340

Etymology: the epithet “*globulosus*” refers to pileipellis terminal hyphae inflated to spherical to prolate.

Diagnosis: This species is distinguished from closed species by a convex to applanate pileus that is deep reddish brown at the center, lighter-colored outwards, and light yellow margin, striped; white to light yellowish green lamellae, with a reddish brown and smooth stipe, pileipellis two layers and the terminal hyphae inflated to spherical to prolate, and a bit bigger basidiospores.

Type: China. Jilin Province: Tonghua City, Ji′an County, Wunvfeng National Forest Park, 41.28° N, 126.14° E, 28 August 2019, Yong-Lan Tuo and Jia-Jun Hu, HMJAU 60307, holotype (GenBank accession no.: ITS = OM030269, nLSU = OM033406).

Basidiomata medium-sized, gregarious. Pileus convex to applanate, 4.5–5.5 cm diameter, smooth, deep reddish brown at the center, yellowish brown towards margin; margin white to light yellow, striped, entire, wavy. Context thin, fleshy, brown, odorless. Stipe center, clavate, 4.8–6.0 cm long and 0.6–0.8 cm wide, deep reddish brown, paler at apex, smooth, fistulose, fibrous. Lamellae adnexed to adnate, white to light yellowish green, I = 1–3, L = 9–15, crowded.

Basidiospores elliptic, (6.8) 7.0–8.8 (9.0) × (3.1) 3.3–4.2 (4.8) µm, Q = (1.63) 1.75–2.20 (2.26), Qm = 1.93 ± 0.16, smooth, hyaline, inamyloid, thin-walled. Basidia clavate, (23) 25–32 (33) × 6–9 (11) µm, two- or four-spored, thin-walled, smooth, hyaline. Cheilocystidia abundant, clavate, with obtuse on the top, (22) 24–38(39) × 5–9 (10) µm, thin-walled, smooth, hyaline. Pileipellis layered, the upper layer inflated to spherical to prolate hyphae, 15–33 (47) µm wide, brown, smooth, thin-walled; down layer made up of branched and inflated hyphae, pigment light brown to brown incrusting in pileipellis, thin-to-thick-walled.

Ecology: Grows on rotten wood.

Distribution: China (Jilin Province)

Other specimen examined: China. Jilin Province: Tonghua City, Ji’an County, Wunvfeng National Forest Park, 41.28° N, 126.14° E, 3 September 2021, Yong-Lan Tuo and Jia-Jun Hu, HMJAU 60308 (GenBank Accession no.: ITS = OM030270, nLSU = OM033407).

Note: In terms of morphology, *Gymnopus globulosus* resembles *Gymnopus erythropus* and *Gymnopus tiliicola* in its red to dark red pileus and stipe. However, *Gymnopus globulosus* is distinguishable from *Gymnopus erythropus* due to its deeper-colored pileus, light yellowish green lamellae, which is light yellow of *Gymnopus erythropus.* In terms of microfeature, the pileipellis of *Gymnopus erythropus* is between a cutis and a trichoderm, while the pileipellis of *Gymnopus globulosus* is layered, with the upper layer inflated to spherical to prolate hyphae and the down layer made up of branched and inflated hyphae, and bigger basidiospores. *Gymnopus globulosus* differs from *Gymnopus tiliicola* with its deeper-colored pileus, light yellowish green lamellae, grows on rotten wood, pileipellis two layers and the terminal hyphae inflated to spherical to prolate, and bigger basidiospores.

New record from Jilin Province, China

*Gymnopus erythropus* (Pers.) Antonín, Halling and Noordel.

**Figure 12:**
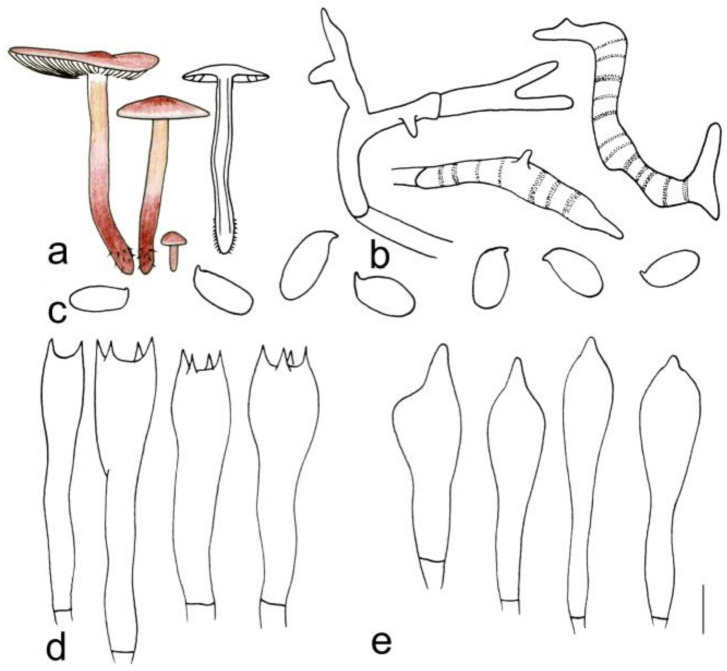
Morphological characteristics of *Gymnopus erythropus* (HMJAU 60315). (**a**) Basidomata, (**b**) pileipellis, (**c**) basidiospores, (**d**) basidia, and (**e**) cheilocystidia. Scale bars: 1 cm (**a**), 25 µm (**b**), and 5 µm (**c**–**e**).

Figure 3h and Figure 12

Basidiomata small-to-medium-sized, scattered to gregarious. Pileus convex to applanate, 1.1–3.2 cm diameter, smooth, hygrophanus, reddish brown to brown at the center, light reddish brown to yellowish brown towards margin; margin beige to light yellow, entire, wavy sometimes. Context thin, fleshy, light brown, odorless. Stipe center, cylindrical, 4.1–10.0 cm long and 0.2–0.5 cm wide, deep reddish brown to light reddish brown, paler at apex, smooth, covered with scattered light yellow to brown hairs hairy at base, fistulose, fibrous. Lamellae adnate, fresh-pink, I = 3–5, L = 14–27, crowded.

Basidiospores elliptic, (5.0) 6.0–8.2 (10.0) × (2.1) 3.0–5.0 (6.0) µm, Q = (1.20) 1.48–2.33 (3.00), Qm = 1.87 ± 0.27, smooth, hyaline, inamyloid, thin-walled. Basidia clavate, (17) 21–33 (38) × (4) 5–9 (10) µm, two- or four-spored, thin-walled, smooth, hyaline. Cheilocystidia abundant, clavate, with obtuse on the top, (15)21–33 (39) × (3) 4–8 (9) µm, thin-walled, smooth, hyaline. Pileipellis a cutis, made up of irregular branched or weakly coralloid hyphae, inflated, (6) 8–20 (20) µm wide, hyaline to light yellow, smooth, thin-walled, clamps present. 

Ecology: Grows on the deciduous layer or rotten branches in coniferous and broad-leaved mixed forest.

Distribution: China (Jilin Province)

Specimen examined: China. Jilin Province: Baishan City, Changbai Korean Autonomous County, Wangtian’e Scenic Spot, 41.56° N, 127.95° E, 8 September 2019, Jia-Jun Hu, Gui-ping Zhao, and Bo Zhang, HMJAU 60309; Baishan City, Changbai Korean Autonomous County, Wangtian’e Scenic Spot, 41.56° N, 127.95° E, 8 September 2019, Jia-Jun Hu, Gui-ping Zhao, and Bo Zhang, HMJAU 60315 (GenBank Acc. no.: ITS = OM030280, nLSU = OM033395); Baishan City, Fusong County, Lushuihe Town, 42.53° N, 127.80° E, 8 September 2019, Jia-Jun Hu, Gui-ping Zhao, and Bo Zhang, HMJAU 60313 (GenBank Acc. no.: ITS = OM030281, nLSU = OM033396); Yanbian Korean Autonomous Prefecture, Antu County, Edaobaihe Town, 42.39° N, 128.11° E, 4 September 2018, Jia-Jun Hu and Bo Zhang, HMJAU 60310; HMJAU 60311; HMJAU 60312; Liaoning Province: Jinzhou City, Yi County, Mt. Yiwulv, 24 September 2013, Di Wang, HMJAU 28892; Jinzhou City, Yi County, Mt. Yiwulv, 25 September 2013, Di Wang, HMJAU 28839.

**Table 2:** Macrocharacteristics comparison between our new species, *Gymnopus* *erythropus* and *Gymnopus* *fagiphilus*.

Scientific Name	Pileus	Lamellae	Stipe
*G. longisterigmaticus*	1.5–3.2 cm diameter, smooth, brown at center, light brown to yellow towards the margin, margin light yellow to yellowish white, entire	Lamellae subfree to adnate, white to light yellow, I = 1–3, L = 15–18, crowded	3.2–5.0 × 0.2–0.3 cm, reddish brown, smooth, covered with white to light reddish brown density hairs at base
*G. longus*	1.7–3.7 cm diameter, smooth, reddish brown at center, towards margin light reddish brown to brown; margin white to light yellow or light brown	Adnate, white to light yellow, I = 5–7, L = 19–24, crowded	3.7–4.3 cm × 0.3–0.6 cm, reddish brown, with brown farinose on the upper part, and white to light reddish brown tomentose at the base
*G. macrosporus*	1.2–4.6 cm diameter, smooth, deep reddish brown at center, reddish brown to yellowish brown towards margin; margin beige to light yellow, striped, wavy	Adnexed to adnate or near free, light yellow, I = 1–3, L = 13–17, crowded	7.8–9.5 × 0.2–0.5 cm, deep reddish brown to reddish brown, smooth, and light yellow to light reddish brown tomentose at the base
*G. striatus*	Depressed when old, 2.3–4.1 cm diameter, smooth, cinnamon at center, brown to light brown towards margin; margin white to light yellow, striped, wavy	Adnate, yellow to light brown, I = 3–9, L = 17–23, crowded	5.5–7.0 cm × 0.3–0.8 cm, deep reddish brown to reddish brown, longitudinal striped, covered with yellow to light brown hairs up to 1/3 (from the base upwards), fistulose, fibrous
*G. changbaiensis*	2.1–3.4 cm diameter, smooth, hygrophanus, reddish brown at the center, light pink towards margin; margin white to light pink, striped	Adnate, fresh to pink, I = 1–5, L = 19–24, crowded	4.2–5.3 cm × 0.2–0.3 cm, deep reddish brown to reddish brown, covered with light yellow to brown hairs up to 1/3 (from the base upwards)
*G. tomentosus*	1.6–3.0 cm diameter, smooth, tan at the center, light brown towards margin; margin white, tomentose	Adnexed, yellowish green, I = 3–7, L = 19–25, crowded	3.3–4.3 cm × 0.2–0.5 cm, blackish green at apex, reddish brown below, covered with reddish brown hairs up to 1/4 (from the base upwards)
*G. tiliicola*	3.0–6.7 cm diameter, smooth, deep rose-red at the center, yellowish pink towards margin; margin white to light yellow, striped	Adnexed to adnate, light pink to fresh, I = 1–3, L = 19–24, crowded	2.2–4.5 cm × 0.3–0.7 cm, deep reddish brown, smooth
*G. globulosus*	4.5–5.5 cm diameter, smooth, deep reddish brown at the center, yellowish brown towards margin; margin white to light yellow, striped	Adnexed to adnate, white to light yellowish green, I = 1–3, L = 9–15, crowded	4.8–6.0 cm × 0.6–0.8 cm, deep reddish brown, paler at apex, smooth
*G. erythropus*	1.1–3.2 cm diameter, smooth, hygrophanus, reddish brown to brown at the center, light reddish brown to yellowish brown towards margin; margin beige to light yellow	Adnate, fresh-pink, I = 3–5, L = 14–27, crowded	4.1–10.0 cm × 0.2–0.5 cm, deep reddish brown to light reddish brown, paler at apex, smooth, covered with scattered light yellow to brown hairs hairy at base
*G. fagiphilus*	(7) 15–25 (35) mm broad, when moist slightly translucently striate at margin, yellow-brown or reddish brown, paler at margin	Moderately distant, L = 18–21, I = 3–7 (15), free or narrowly adnate, rarely adnate, pinkish brown or pinkish cream, darker with age or tinged gray	20–40 (70) × 1–3 mm, orange-brown to red-brown, sometimes paler at apex, dark red-brown towards base, covered with fine, white or yellow hairs up to 2/3 of length (from base upwards)

Note: The description of *Gymnopus fagiphilus* is based on Antonín and Noordeloos [1].

**Table 3:** Microcharacteristics comparison between our new species, *Gymnopus erythropus* and *Gymnopus fagiphilus*.

Scientific Name	Pileipellis	Q	Qm	Basidiospores	Basidia	Cheilocystidia	Caulocystidia
*G. longisterigmaticus*	Cutis, irregularly branched hyphae, inflated, 10–27 (35) µm wide, hyaline to light yellow, smooth or pigment yellowish brown incrusting in pileipellis	(1.40) 1.67–2.25 (2.26)	1.93 ± 0.20	(6.2) 6.7–9.0 × (3.0) 3.1–4.3 (5.0) μm	Clavate, (18) 19–27 (28) × (5) 6–10 µm, four-spored; sterigmata extremely long, up to 40 µm	Clavate, with obtuse on the top, or branched, (16) 18–27 × (4) 5–8 (9) µm	None
*G. longus*	A translation between a cutis and a trichoderm, made up of irregularly interwoven, repent or ascending inflated hyphae with inflated and irregularly branched terminal elements, hyaline to light brown, (6) 7–13 (15) µm wide, smooth or pigment yellowish brown incrusting in pileipellis	(1.27) 1.47–2.19 (2.58)	1.80 ± 0.24	(5.6) 6.0–8.0 × (3.0)3.1–4.1 (4.9) μm	(19) 20–28 (29) × 6–9 µm, two- or four-spored, clavate; sterigmata extremely long, up to 33 µm long	Clavate, (21) 22–29 (30) × 5–7 µm, with obtuse on the top	None
*G. macrosporus*	Cutis, made up of irregular branched or weakly coralloid hyphae, inflated, 10–27 (35) µm wide, hyaline to light yellow, smooth	(1.63) 1.67–2.32 (2.37)	1.88 ± 0.18	(6.0) 6.8–7.9 (8.3) × (3.0) 3.1–4.2 (4.3) μm	Clavate, 20–29 × 6–9 µm, two- or four-spored, thin-walled; sterigmata extremely long, up to 32 µm	Clavate, with obtuse on the top, 20–28 (30) × 5 (6)–9 µm	None
*G. striatus*	Cutis, made up of irregular branched or weakly coralloid hyphae, inflated, 10–30 (35) µm wide, hyaline to light yellow, smooth	(1.50) 1.58–2.50 (2.60)	2.01 ± 0.25	6.0–8.0 (9.0) × 3.0–4.0 μm	Clavate, 20 (21)–34 (37) × 5–10 µm, two- or four-spored	Clavate, with obtuse on the top, (17) 20–30 × 4–8 (10) µm	None
*G. changbaiensis*	Cutis, made up of irregular branched or weakly coralloid hyphae, inflated, 8–23 (25) µm wide, hyaline to light yellow	(1.41) 1.53–2.40 (2.50)	1.98 ± 0.24	(5.8) 6.0–8.1 (9.0) × 3.0–4.1(4.2) μm	Clavate, (19) 20–29 (32) × 5–8 µm, two- or four-spored	Clavate, with obtuse on the top, (23) 24–34 (39) × (5) 6–7 (9) µm	None
*G. tomentosus*	Cutis, made up of irregular branched to weakly coralloid or bulbous hyphae, inflated, 10–18 (21) µm wide, light brown	(1.50) 1.59–2.33 (2.40)	1.92 ± 0.23	(6.0) 6.2–8.2 (9.0) × 3.0–4.1 (4.2) μm	Clavate, 20–30 (31) × 5–8 µm, two- or four-spored	Clavate, with obtuse on the top sometimes, (20) 22–30 (32) × 5–7 µm	None
*G. tiliicola*	Cutis, made up of irregular branched to weakly coralloid hyphae, inflated, (5)6–15(17) µm wide, light brown, smooth	(1.70) 1.75–2.26 (2.33)	1.93 ± 0.17	(6.0) 6.9–8.0 (8.2) × (3.0) 3.1–4.0 (4.2) μm	Clavate, 20–30 × 6–8 µm, two- or four-spored	Clavate, with obtuse on the top, (20) 21–27 (28) × 5–7 µm	None
*G. globulosus*	Layered, the upper layer inflated to spherical to prolate hyphae, 15–33 (47) µm wide, brown, smooth, thin-walled; down layer made up of branched and inflated hyphae, pigment light brown to brown incrusting in pileipellis, thin-to-thick-walled	(1.63) 1.75–2.20 (2.26)	1.93 ± 0.16	(6.8) 7.0–8.8(9.0) × (3.1) 3.3–4.2 (4.8) μm	Clavate, (23) 25–32 (33) × 6–9 (11) µm, two- or four-spored	Clavate, with obtuse on the top, (22) 24–38 (39) × 5–9 (10) µm	None
*G. erythropus*	Cutis, made up of irregular branched or weakly coralloid hyphae, inflated, (6) 8–20 (20) µm wide, hyaline to light yellow, smooth	(1.20) 1.48–2.33 (3.00)	1.87 ± 0.27	(5.0) 6.0–8.2 (10.0) × (2.1) 3.0–5.0 (6.0) µm	Clavate, (17) 21–33 (38) × (4) 5–9 (10) µm, two- or four-spored	Clavate, with obtuse on the top, (15) 21–33 (39) × (3) 4–8 (9) µm, thin-walled, smooth, hyaline	None
*G. fagiphilus*	Cutis with transitions to a trichoderm, made up of irregularly shaped, 4.0–15 (25) μm–wide coralloid elements (“Dryophila-structure”); pigment brown-yellow, incrusting in pileipellis	1.7–2.3	2.1	(6.0) 7.0–9.0 × (3.0) 3.5–4.5 μm	21–31 × 6.0–8.5 μm, 4-spored	15–40 (60) × 4.0–8.0 (10) μm, irregularly clavate, often with lobed apex or with short to long, up to 10 μm long rostrum, sometimes very slender lageniform	20–80 (120) × 4.0–12 μm, subcylindrical or sublageniform, numerous

Note: The description of *Gymnopus fagiphilus* is based on Antonín and Noordeloos [1].

Key to the species reported in this study

1 Stipe covered with dense hairs at the base··············································································2

1 Stipe smooth, or covered with sparse hairs at the base ························*Gymnopus erythropus*

2 Basidia sterigmata extremely long···························································································3

2 Basidia sterigmata short·············································································································5

3 Stipe smooth in upper part········································································································4

3 Stipe covered with brown farinose on the upper part·································*Gymnopus longus*

4 Pileus pale color, stipe color uneven···········································*Gymnopus longisterigmaticus*

4 Pileus dark color, stipe color uniform·············································*Gymnopus macrosporus*

5 Growing on the deciduous layer or rotten branches·····························································6

5 Grows at the base of *Tilia* sp.··········································································*Gymnopus tiliicola*

6 Pileus pale color, near white·································································*Gymnopus tomentosus*

6 Pileus deep color·······················································································································7

7 Stipe covered with longitudinally stripes····················································*Gymnopus striatus*

7 Stipe without longitudinally stripes·························································································8

8 Pileipellis a cuits, typically “*Dryophila* type”·····································*Gymnopus changbaiensis*

8 Pileipellis layered, hyphae inflated to spherical to prolate···················*Gymnopus globulosus*

## 4. Discussion

### 4.1. New Sights on Morphological Characteristics

The genus *Gymnopus* is geographically widely distributed; however, in China, its species diversity is poorly known. Moreover, in China, only three species were originally described with molecular evidence. One of these is *Gymnopus ramulicola* T.H. Li and S.F. Deng [27] from Hainan Province, China; the second one is *Gymnopus alliifoetidissimus* T.H. Li and J.P. Li [25] from Guangdong Province, China; and the third is *Gymnopus pallipes* J.P. Li and Chun Y. Deng [25] from Guangdong and Guizhou Province, China. In our study, eight new species of *Gymnopus* from China are described as new species. They are well-supported by molecular phylogenetic and morphological evidence. Our newly recognized and delimited species are distributed in the broad-leaved and mixed forests, and occur in early autumn in Northeast China. The species we described here are hardly seen in the wild mushroom market; thus, their edibility is not yet known.

The description of these new species also broadens the morphological characterization of the genus *Gymnopus*. In the previous study, the pileipellis of the species in this genus was a cuits to trichoderm. Moreover, the pileipellis in the species of sect. *Levipedes* was an entangled, not radially oriented trichoderm of inflated, often lobed or coralloid elements of the “*Dryophila* type” [1,17]. In this study, the pileipellis of *Gymnopus globulosus* was divided into two layers, with the upper layer comprising hyphae inflated to spherical to prolate, differing from that of all known species in the genus, while the second layer was typical of the “*Dryophila* type”. To our knowledge, the sterigmata of the basidia are usually not too long; however, the species *Gymnopus longistrigmaticus*, *Gymnopus longus*, and *Gymnopus macrosporus* had extremely long sterigmata, up to 40 μm. Thus, the structure of extremely long basidia sterigmata is traceable in our species. In addition, all the species described from this study are detailed compared in macro- and micro-features (Table 2 and Table 3).

### 4.2. Phylogenetic Relationships of Gymnopus s.l. with Related Genera

Phylogenetic analyses of the species of *Gymnopus* s.l. and the related genera presented in this study confirmed that the genus *Gymnopus* defined by Antonín and Noordeloos, as well as Halling, is not monophyletic in a strongly supported clade. Similar results were observed with our phylogenetic analysis. Our results, thus, support the finding of Oliveira et al., promoting sect. *Perforanita* to the genus level, *Paragymnopus*, and share a close affinity with *Lentinula*. Moreover, sect. *Vestipedes* was clearly separated from *Gymnopus* s. str [12,15], and were closed to *Marasmiellus*, *Collybiopsis*, and *Rhodocollybia*. However, in their study, the species of *Gymnopus* sect. *Vestipedes* was involved with *Marasmiellus*; therefore, Oliveira et al. [15] proposed to transfer *Gymnopus* sect. *Vestipedes* to *Marasmiellus* and redefined the genus *Gymnopus* more strictly. 

However, in our phylogenetic analyses, a different result was obtained. In our results, sect. *Vestipedes* did not group into one clade with *Marasmiellus* to form an independent clade, forming a sister clade to genus *Collybiopsis*. Furthermore, the taxonomic status of *Collybiopsis minor* R.H. Petersen still needs to be clarified; in our study, *C. minor* was separated far away from *Collybiopsis*, while being clustered with sect. *Vestipedes* within a single clade. 

Some species of sect. *Vestipedes* and genus *Marasmiellus* have been proposed for transfer to other genera in recent years. *Gymnopus cylindricus* J.L. Mata and *Gymnopus brunneigracilis* (Corner) A.W. Wilson, Desjardin and E. Horak were suggested to be switched into *Marasiellus*. The type species of *Marasmiellus*, *Marasmiellus juniperinus*, and some other species within the genus, were advised to be relocated to *Collybiopsis* [47]. Thus, the boundaries between *Gymnopus*, *Marasmiellus,* and *Collybiopsis* would be more blurred, especially between *Marasmiellus* and *Collybiopsis*, as well as if these species were transferred to *Collybiopsis*; then it would be multiphyletic, with *Rhodocollybia*, *Paragymnopus*, and *Lentinula* would becoming synonyms of *Gymnopus*.

### 4.3. Nova Suggestions of Phylogenetic Relationships within Gymnopus s. str.

In our phylogenetic results, the genus *Gymnopus*, which was defined by Oliveira et al. [15], was mainly divided into four clades. Sect. *Levipedes*, sect. *Gymnopus*, and sect. *Androsacei* are somewhat more closely related, whereas they are distant from the sect. *Impudicae*. Before 2010, both sect. *Impudicae* and sect. *Levipedes* were subsections below the same section. However, from our results, sect. *Impudicae* and sect. *Levipedes* are more distantly related, probably due to similar environments, causing a similar appearance. In addition, the genus *Mycetines* and sect. *Impudicae*, with a strong odor, are not closely related to each other, and this is consistent with the result that they have a different pileipellis structure of pileus.

Thus, sect. *Levipedes* being split into two sections was supported by the phylogenetic analysis. Sect. *Levipedes* subsect. *Levipedes* was also divided into two subclades: one is *Gymnopus dryophilus* complex, a subclade (defined here as/*dryophila*) that includes all the *Gymnopus dryophilus* complex species reported around the world (characterized by a *Gymnopus dryophilus*–like appearance and arises in early spring or later in the autumn). From the result, the East Asia sequences of *Gymnopus dryophilus* were not clustered with the European sequences, while they were clustered with the new species—*Gymnopus dryophiloides*—that Antonín, Ryoo and Ka reported from Korea in 2020. Antonín et al. [51] do not accept *Gymnopus lanipes* (Malençon and Bertault) Vila and Llimona as a separate species and consider it to be a variant of *Gymnopus dryophilus*. From our phylogenetic result, it is clear that *Gymnopus lanipes* clusters with *Gymnopus inexpectatus*, which Consiglio, Vizzini, Antonín and Contu described from Europe, which, if *Gymnopus lanipes* is not considered an independent species, then *Gymnopus dryophioides* and *Gymnopus inexpectatus* should equally be treated as *Gymnopus dryophilus.* Moreover, *Gymnopus erythropus* complex, a subclade (defined here as/*erythropus*), includes *Gymnopus erythropus*, *Gymnopus fagiphilus,* and our new species (characterized by a red to reddish brown color, a smooth or scattered-to-dense tomentose stipe, and occurring in early autumn). The above results imply the need for a deeper and more extensive study on sect. *Levipedes.*

Based on the current study’s findings, we increased the species diversity of the genus *Gymnopus* from China. However, probably due to the lacking of species sampling or the inadequate genetic variation in the DNA loci in our study, the deep phylogenetic relationships within the genus *Gymnopus* and between the related genera—*Lentinula*, *Rhodocollybia*, *Mycetinis*, *Collybiopsis*, etc.—remain unresolved. Thus, in future work, more species of this genus and similar genera will be discovered, which will provide new evidence and, thus, lead to a deeper understanding of the relationships within and among these genera.

## Data Availability

Not applicable.

## References

[1] Antonín V., Noordeloos M.E. (2010). A Monograph of Marasmioid and Collybioid Fungi in Europ.

[2] Persoon C.H. (1801). Synopsis Methodica Fungorum 1.

[3] Fries E.M. (1821). Systema Mycologicum: Sistens Fungorum Ordines, Genera Et Species, Huc Usque Cognitas.

[4] Staude F. (1857). Die Schwämme Mitteldeutschlands, Insbesondere Des Herzogtums Coburg.

[5] Singer R. (1962). The Agaricales in Modern Taxonomy.

[6] Singer R. (1975). The Agaricales in Modern Taxonomy.

[7] Singer R. (1986). The Agaricales in Modern Taxonomy.

[8] Halling R.E. (1983). The Genus Collybia (Agaricales) in the Northeastern United States and Adjacent Canada. Mycol. Mem..

[9] Antonín V., Noordeloos M.E. (1993). A Monograph of Marasmius, Collybia and Related Genera in Europe. Part 1: Marasmius, Setulipes, and Marasmiellus.

[10] Antonín V., Noordeloos M.E. (1997). A Monograph of Marasmius, Collybia and Related Genera in Europe. Part 2: Collybia, Gymnopus, Rhodocollybia, Crinipellis, Chaetocalathus, and Additions to Marasmiellus.

[11] Antonín V., Halling R., Noordeloos M. (1997). Generic Concepts within the Groups of Marasmius and Collybia Sensu Lato. Mycotaxon.

[12] Wilson A.W., Desjardin D.E. (2005). Phylogenetic Relationships in the Gymnopoid and Marasmioid Fungi (Basidiomycetes, Euagarics Clade). Mycologia.

[13] Moncalvo J.M., Vilgalys R., Redhead S.A., Johnson J.E., James T.Y., Catherine Aime M., Hofstetter V., Verduin S.J.W., Larsson E., Baroni T.J. (2002). One Hundred and Seventeen Clades of Euagarics. Mol. Phylogenet. Evol..

[14] Mata J.L., Hughes K.W., Petersen R.H. (2004). Phylogenetic Placement of Marasmiellus juniperinus. Mycoscience.

[15] Oliveira J.J., Vargas-Isla R., Cabral T.S., Rodrigues D.P., Ishikawa N.K. (2019). Progress on the Phylogeny of the Omphalotaceae: Gymnopus S. Str., Marasmiellus S. Str., Paragymnopus gen. nov. and Pusillomyces gen. nov. Mycol. Prog..

[16] Petersen R.H., Hughes K.W. (2020). Two New Genera of Gymnopoid/Marasmioid Euagarics. Mycotaxon.

[17] Halling R.E. (1996). Notes on Collybia V. Gymnopus Section Levipedes in Tropical South America, with Comments on Collybia. Brittonia.

[18] Singer R. (1961). Type Studies in Basidiomycetes. X. Pers. Mol. Phylogeny Evol. Fungi.

[19] Jansen A. (1991). Het Geslacht Collybia (De Fungi Van Nederland).

[20] Kirk P., Cannon P., Minter D., Stalpers J. (2008). Dictionary of the Fungi.

[21] Teng S.Q. (1963). Fungi of China.

[22] Tai F.L. (1979). Sylloge Fungorum Sinicorum.

[23] Deng S.F. (2016). Taxonomy of Gymnopus and Preliminary Study of Marasmiaceae Resource in South China. Master’s Thesis.

[24] Li J.-P., Song B., Feng Z., Wang J., Deng C.-Y., Yang Y.-H. (2021). A New Species of Gymnopus Sect. Androsacei (Omphalotaceae, Agaricales) from China. Phytotaxa.

[25] Li J.-P., Li Y., Li T.-H., Antonín V., Hosen I., Song B., Xie M.-L., Feng Z. (2021). A Preliminary Report of Gymnopus Sect. Impudicae (Omphalotaceae) from China. Phytotaxa.

[26] Mešić A., Tkalčec Z., Deng C.-Y., Li T.-H., Pleše B., Ćetković H. (2011). Gymnopus Fuscotramus (Agaricales), a New Species from Southern China. Mycotaxon.

[27] Deng S.-F., Li T.-H., Jiang Z.-D., Song B. (2016). Gymnopus Ramulicola sp. nov., a Pinkish Species from Southern China. Mycotaxon.

[28] Wang X.S., Bao J.S., Bao H., Feng J. (2020). Macrofungal Diversity in Hanwula National Nature Reserve, Inner Mongolia. Mycosystema.

[29] Sun Y.L., Luo Y., Bau T. (2021). Notes on Basidiomycetes of Jilin Province (X). J. Fungal Res..

[30] Royal Botanic Garden E. (1969). Flora of British Fungi: Colour Identification Chart.

[31] César E., Bandala V.M., Montoya L., Ramos-Fernández A. (2018). A New Gymnopus Species with Rhizomorphs and Its Record as Nesting Material by Birds (Tyrannideae) in the Subtropical Cloud Forest from Eastern Mexico. MycoKeys.

[32] Gardes M., Bruns T.D. (1993). Its Primers with Enhanced Specificity for Basidiomycetes-Application to the Identification of Mycorrhizae and Rusts. Mol. Ecol..

[33] Cubeta M.A., Echandi E., Abernethy T., Vilgalys R. (1991). Characterization of Anastomosis Groups of Binucleate Rhizoctonia Species Using Restriction Analysis of an Amplified Ribosomal Rna Gene. Phytopathology.

[34] Vilgalys R., Hester M. (1990). Rapid Genetic Identification and Mapping of Enzymatically Amplified Ribosomal DNA from Several Cryptococcus Species. J. Bacteriol..

[35] Coimbra V.R.M., Pinheiro F.G.B., Wartchow F., Gibertoni T.B. (2015). Studies on Gymnopus Sect. Impudicae (Omphalotaceae, Agaricales) from Northern Brazil: Two New Species and Notes on G. Montagnei. Mycol. Prog..

[36] Ryoo R., Antonín V., Ka K.-H., Tomšovský M. (2016). Marasmioid and Gymnopoid Fungi of the Republic of Korea. 8. Gymnopus Section Impudicae. Phytotaxa.

[37] Thonpson J. (1997). The Clustal X Windows Interface: Flexible Strategies for Multiple Sequence Alignment Aided by Quality Analysis Tools. Nucl. Acids Res..

[38] Ranwez V., Douzery E.J.P., Cambon C., Chantret N., Delsuc F. (2018). Macse V2: Toolkit for the Alignment of Coding Sequences Accounting for Frameshifts and Stop Codons. Mol. Biol. Evol..

[39] Katoh K., Standley D.M. (2013). Mafft Multiple Sequence Alignment Software Version 7: Improvements in Performance and Usability. Mol. Biol. Evol..

[40] Hall T. (1999). Bioedit: A User-Friendly Biological Sequence Alignment Editor and Analysis Program for Windows 95/98/Nt. Nucleic Acids Symp. Ser..

[41] Zhang D., Gao F., Jakovlić I., Zhou H., Zhang J., Li W.X., Wang G.T. (2020). Phylosuite: An Integrated and Scalable Desktop Platform for Streamlined Molecular Sequence Data Management and Evolutionary Phylogenetics Studies. Mol. Ecol. Resour..

[42] Kalyaanamoorthy S., Minh B.Q., Wong T.K.F., Von Haeseler A., Jermiin L.S. (2017). Modelfinder: Fast Model Selection for Accurate Phylogenetic Estimates. Nat. Methods.

[43] Ronquist F., Huelsenbeck J.P. (2003). Mrbayes 3: Bayesian Phylogenetic Inference under Mixed Models. Bioinformatics.

[44] Edler D., Klein J., Antonelli A., Silvestro D. (2021). Raxmlgui 2.0: A Graphical Interface and Toolkit for Phylogenetic Analyses Using Raxml. Methods Ecol. Evol..

[45] Vizzini A., Antonín V., Sesli E., Contu M. (2015). Gymnopus Trabzonensis sp. nov. Omphalotaceae and Tricholoma Virgatum var. Fulvoumbonatum var. nov. Tricholomataceae, Two New White-Spored Agarics from Turkey. Phytotaxa.

[46] Mata J.L., Hughes K.W., Petersen R.H. (2006). An Investigation of/Omphalotaceae (Fungi: Euagarics) with Emphasis on the Genus Gymnopus. SYDOWIA-HORN-.

[47] Petersen R.H., Hughes K.W. (2021). Collybiopsis and Its Type Species, Co. Ramealis. Mycotaxon.

[48] Wilson A., Desjardin D., Horak E. (2004). Agaricales of Indonesia: 5. The Genus Gymnopus from Java and Bali. Sydowia.

[49] Schoch C.L., Robbertse B., Robert V., Vu D., Cardinali G., Irinyi L., Meyer W., Nilsson R.H., Hughes K., Miller A.N. (2014). Finding Needles in Haystacks: Linking Scientific Names, Reference Specimens and Molecular Data for Fungi. Database.

[50] Petersen R.H., Hughes K.W. (2016). Micromphale Sect. Perforantia (Agaricales, Basidiomycetes); Expansion and Phylogenetic Placement. MycoKeys.

[51] Antonín V., Sedlák P., Tomšovský M. (2013). Taxonomy and Phylogeny of European Gymnopus Subsection Levipedes (Basidiomycota, Omphalotaceae). Persoonia.

[52] Vu D., Groenewald M., De Vries M., Gehrmann T., Stielow B., Eberhardt U., Al-Hatmi A., Groenewald J.Z., Cardinali G., Houbraken J. (2019). Large-Scale Generation and Analysis of Filamentous Fungal DNA Barcodes Boosts Coverage for Kingdom Fungi and Reveals Thresholds for Fungal Species and Higher Taxon Delimitation. Stud. Mycol..

[53] Ryoo R., Antonín V., Ka K.H. (2020). Marasmioid and Gymnopoid Fungi of the Republic of Korea. 8. Gymnopus Section Levipedes. Mycobiology.

[54] Vizzini A., Consiglio G., Antonin V., Contu M. (2008). A New Species within the Gymnopus Dryophilus Complex (Agaricomycetes, Basidiomycota) from Italy. Mycotaxon.

[55] Mata J.L., Petersen R.H., Hughes K.W. (2001). The Genus Lentinula in the Americas. Mycologia.

[56] Hibbett D.S., Hansen K., Donoghue M.J. (1998). Phylogeny and Biogeography of Lentinula Inferred from an Expanded Rdna Dataset. Mycol. Res..

[57] Antonín V., Ryoo R., Shin H.-D. (2010). Marasmioid and Gymnopoid Fungi of the Republic of Korea. 2. Marasmius Sect. Globulares. Persoonia.

[58] Petersen R.H., Hughes K.W. (2017). An Investigation on Mycetinis (Euagarics, Basidiomycota). MycoKeys.

[59] Mata J.L., Ovrebo C.L., Baroni T.J., Hughes K.W. (2016). New Species of Neotropical Rhodocollybia. Mycotaxon.

